# Network and pairwise meta-analysis of the effects of novel hypoglycemic agents on cardiorenal outcomes in patients with type 2 diabetes and heart failure

**DOI:** 10.3389/fendo.2026.1955431

**Published:** 2026-10-14

**Authors:** Xuejiao Ye, Qian Wu, Leyang Li, Mingyu Huang, Shihan Wang

**Affiliations:** China Academy of Chinese Medical Sciences, Guang’anmen Hospital, Beijing, China

**Keywords:** dipeptidyl peptidase-4 inhibitor, glucagon-like peptide-1 receptor agonist, glucose-dependent insulinotropic polypeptide/glucagon-like peptide-1 dual receptor agonist, heart failure, sodium-glucose cotransporter-2 inhibitor, type 2 diabetes mellitus

## Abstract

**Objective:**

This study aims to evaluate the cardiovascular and renal benefits of novel hypoglycemic agents, including glucagon-like peptide-1 receptor agonists (GLP-1RAs), dipeptidyl peptidase-4 inhibitors (DPP-4is), sodium-glucose cotransporter-2 inhibitors (SGLT-2is), and glucose-dependent insulinotropic polypeptide/glucagon-like peptide-1 dual receptor agonists (GIP/GLP-1RAs) in patients with type 2 diabetes mellitus (T2DM) and heart failure (HF).

**Methods:**

Systematic literature searches were conducted in MEDLINE, Embase, ClinicalTrials.gov, and Cochrane Central Register of Controlled Trials (CENTRAL) from inception to 30 April 2026. Randomized controlled trials (RCTs) for cardiovascular outcomes (CVOTs) or *post hoc* analyses of CVOTs comparing SGLT-2is, DPP-4is, GLP-1RAs, or GIP/GLP-1RAs in patients with T2DM and HF were selected. Network meta-analysis was used for indirect comparisons, with results presented as hazard ratios (HRs) and 95% confidence intervals (CIs).

**Results:**

A total of 10 RCTs and two *post hoc* analyses of CVOTs involving 13,886 patients were included. Compared with placebo, SGLT-2is (HR [95% CI]: 0.75 [0.68, 0.82]) and GLP-1RAs (HR [95% CI]: 0.77 [0.61, 0.97]) both significantly reduced the risk of HF-related composite endpoints in patients with T2DM and HF. No statistically significant difference was observed between SGLT-2is and GLP-1RAs (HR [95% CI]: 0.98 [0.76, 1.25]). SGLT-2is also reduced the risk of HF worsening (HR [95% CI]: 0.72 [0.53, 0.96]), HF hospitalization (HR [95% CI]: 0.74 [0.66, 0.83]), and cardiovascular (CV) death (HR [95% CI]: 0.87 [0.77, 0.98]). For all-cause death, SGLT-2is showed a trend toward reduced risk compared to placebo, but this difference did not reach statistical significance (HR [95% CI]: 0.83 [0.67, 1.02]). For the renal composite outcome, SGLT-2is did not demonstrate a statistically significant benefit (HR [95% CI]: 0.76 [0.51, 1.14]).

**Conclusion:**

Optimal clinical management of T2DM requires a comprehensive assessment of cardiovascular and renal comorbidities. This network meta-analysis indicates that SGLT-2is significantly reduce the risk of HF-related composite endpoints and CV death in T2DM patients with HF, although their renoprotective effect does not reach statistical significance. GLP-1RAs also demonstrate beneficial effects on HF-related composite endpoints. These findings suggest that both SGLT-2is and GLP-1RAs are preferred glycemic control strategies for improving cardiovascular outcomes in T2DM patients with HF, while further evidence is needed to confirm their definitive renal benefits.

**Systematic review registration:**

https://www.crd.york.ac.uk/PROSPERO/view/CRD42026142123, identifier: CRD420261421235.

## Introduction

Type 2 diabetes mellitus (T2DM) is a global chronic metabolic disease that independently increases the risk of morbidity and mortality from heart failure (HF). Epidemiological data indicate that there were 589 million adults living with T2DM worldwide in 2024, with an overall prevalence rate of 11.11%. The number is projected to rise to 853 million by 2050, with a prevalence of 12.96%, and the epidemic trend will continue to intensify (1). HF is one of the most common cardiovascular complications of T2DM and is associated with a poor prognosis. Compared with nondiabetic populations, T2DM patients have more than twice the risk of developing HF (2–4), and the prevalence of HF among T2DM patients is as high as 57%. Coexistence of T2DM and HF significantly increases the risk of HF readmission, major adverse cardiovascular events, and all-cause death (5, 6). Traditional hypoglycemic agents fail to exert definite cardiovascular and renal protective effects in T2DM patients with HF, leading to an urgent clinical demand for targeted glucose-lowering strategies that target the bidirectional pathophysiological interaction between metabolic disorder and cardiac injury (7, 8).

The emergence of novel hypoglycemic agents, including sodium-glucose cotransporter-2 inhibitors (SGLT-2is), glucagon-like peptide-1 receptor agonists (GLP-1RAs), glucose-dependent insulinotropic polypeptide/glucagon-like peptide-1 dual receptor agonists (GIP/GLP-1RAs), and dipeptidyl peptidase-4 inhibitors (DPP-4is), has transformed the clinical management of T2DM (9). Large cardiovascular outcomes randomized controlled trials (RCTs) have confirmed that SGLT-2is reduce the risk of HF and renal events in T2DM patients by approximately 18%~25% and 24%~39%, respectively, thereby supporting their role as first-line therapies in patients with cardiorenal comorbidities (10–14). The cardioprotective effects of GLP-1RAs are drug-specific, and whether the benefit represents a class-wide effect remains controversial (15–18). DPP-4is show inconsistent HF risk signals across phase III trials, without unified safety conclusions for HF patients (19–25). Tirzepatide, a novel dual-targeted incretin drug that acts as GIP/GLP-1RAs, shows promising cardiovascular surrogate markers by improving myocardial energy metabolism and reducing cardiac volume load (26–28). Despite being marketed later than the other three classes of drugs, specialized RCTs targeting patients with T2DM and HF remain relatively scarce, and these trials are often characterized by limited sample sizes and short follow-up periods, resulting in insufficient evidence for hard clinical endpoints.

Network meta-analysis, which integrates evidence from direct and indirect comparisons and achieves comprehensive efficacy evaluation through statistical modeling, is an efficient research method to assist clinical decision-making. Even in the absence of head-to-head trials, it can still generate rankings of different interventions. Several previously published meta-analyses have explored the clinical efficacy of various hypoglycemic drugs in patients with T2DM. However, research in patients with T2DM complicated by HF remains significantly limited. Existing studies include a small number of RCTs, have not stratified and synthesized data according to study characteristics, rarely focus on this special high-risk population of patients with T2DM complicated by HF for targeted analysis, and most have not considered GIP/GLP-1RAs as independent study groups. Additionally, rigorous analyses using the Grading of Recommendations Assessment, Development and Evaluation (GRADE) system for standardized grading of outcome evidence quality are lacking. Based on this, this study aims to systematically investigate the effectiveness of novel hypoglycemic drugs, including SGLT-2is, GLP-1RAs, GIP/GLP-1RAs, and DPP-4is, on HF-related composite outcomes, cardiovascular survival outcomes, and renal composite outcomes in patients with T2DM complicated by HF through a network meta-analysis that includes RCTs and cardiovascular outcomes (CVOTs), thereby providing evidence-based support for optimizing clinical hypoglycemic drug selection and improving patient prognosis.

## Methods

### Study registration and reporting

This systematic review was reported in accordance with the Preferred Reporting Items for Systematic Review and Meta-Analyses 2020 (PRISMA) (29). The protocol was prospectively registered on the International Prospective Register of Systematic Reviews database (PROSPERO; registration number: CRD420261421235).

### Search strategy

The search strategy was conducted in accordance with the PRISMA extension statement for network meta-analysis. To identify eligible trials, an electronic search of Medical Literature Analysis and Retrieval System Online (MEDLINE), Cochrane Central Register of Controlled Trials (CENTRAL), Embase, and ClinicalTrials.gov was conducted from inception to 30 April 2026. No time or language restrictions were set. Meanwhile, the reference lists of included studies, previous relevant meta-analyses, and review articles were manually screened to further identify potentially eligible studies. Grey literature, including conference abstracts, was screened, but abstracts without full-text data were excluded per prespecified criteria. Duplicate records were identified using EndNote X9 automated matching and manual cross-checking. The search strategy used terms such as “SGLT-2 inhibitors”, “SGLT-2is”, “DPP-4 inhibitors”, “DPP-4is”, “GLP-1 receptor agonist”, “GLP-1RAs”, “Glucose-dependent Insulinotropic Polypeptide/Glucagon-Like Peptide-1 Receptor Agonist”, “GIP/GLP-1RAs”, “T2DM”, “type 2 diabetes mellitus”, “heart failure”, and “HF” along with their respective abbreviations and synonymous variants. The detailed search strategy for MEDLINE is presented in **Table 1**. The systematic retrieval strategy for Embase is presented in **Supplementary Table S1**, that for CENTRAL in **Supplementary Table S2**, and that for ClinicalTrials.gov in **Supplementary Table S3**.

**Table 1:** Detailed search strategy for MEDLINE.

Search strategy
#1 Diabetes Mellitus, Type 2[MeSH Terms] OR type 2 diabetes[Title/Abstract] OR T2DM[Title/Abstract]
#2 Heart Failure[MeSH Terms] OR heart failure[Title/Abstract] OR congestive heart failure[Title/Abstract]
#3 SGLT2 inhibitor[Title/Abstract] OR sodium-glucose transporter 2 inhibitor[Title/Abstract] OR canagliflozin[Title/Abstract] OR dapagliflozin[Title/Abstract] OR empagliflozin[Title/Abstract] OR ertugliflozin[Title/Abstract]
#4 GLP1 receptor agonist[Title/Abstract] OR glucagon-like peptide 1 receptor agonist[Title/Abstract] OR exenatide[Title/Abstract] OR lixisenatide[Title/Abstract] OR liraglutide[Title/Abstract] OR dulaglutide[Title/Abstract] OR semaglutide[Title/Abstract]
#5 DPP4 inhibitor[Title/Abstract] OR dipeptidyl peptidase 4 inhibitor[Title/Abstract] OR sitagliptin[Title/Abstract] OR vildagliptin[Title/Abstract] OR saxagliptin[Title/Abstract] OR linagliptin[Title/Abstract] OR alogliptin[Title/Abstract] OR teneligliptin[Title/Abstract]
#6 GIP/GLP-1RA[Title/Abstract] OR tirzepatide[Title/Abstract]
#7 #1 AND #2 AND (#3 OR #4 OR #5 OR #6)

### Inclusion criteria

The criteria for including and excluding studies were developed strictly following PICOS principles. The inclusion criteria were as follows: (i) Participants: adults diagnosed with T2DM and HF. (ii) Interventions and comparators: eligible studies reported head-to-head comparisons between any single class of SGLT-2is, DPP-4is, GLP-1RAs, or GIP/GLP-1RAs vs. placebo. (iii) Outcomes: HF-related composite endpoint (including total CV death and hospitalization for HF [first and recurrent]), worsening of HF, hospitalization for HF, all-cause death, cardiovascular (CV) death, and renal composite outcomes (defined as the trial-prespecified adjudicated serious renal composite endpoint from each eligible original RCT. Detailed information is presented in **Supplementary Table S4**: Definition of renal composite endpoint). Multivariable-adjusted hazard ratios (HRs) with corresponding 95% confidence intervals (CIs) were prioritized as effect estimates. (iv) Study design: prospective RCTs for CVOTs or *post hoc* analyses of CVOTs. Exclusion criteria were as follows: (i) duplicate analyses derived from the same trial; (ii) animal experiments, reviews, case reports, editorials, and trials conducted in patients with type 1 diabetes or prediabetes; and (iii) studies that failed to report the outcomes of interest.

### Data extraction

Two researchers (XJ Ye and Q Wu) independently screened abstracts, completed full-text review, and conducted data extraction using EndNote X9. Subsequently, a database was established using Microsoft Excel to record detailed study information, including publication details (title, first author, publication year, trial name, and registration number), patient information (sample size, mean age, gender ratio, comorbidities, and baseline indicators), interventions (medication, dosage, and follow-up duration), outcomes, and study design. To ensure the accuracy of the data, two independent researchers entered the data and cross-checked the data for inconsistencies. For studies with missing data, we contacted the original authors for clarification. If no accurate data were provided, the study was excluded. Any conflicts during this process were resolved through consensus.

### Risk of bias assessment

This study employed two quality assessment tools. For all original RCTs, the Cochrane Risk of Bias Tool 2.0 (RoB 2.0) (29) was used to assess the methodological quality, which includes bias arising from the randomization process, bias due to deviations from intended interventions, bias due to missing outcome data, bias in outcome measurement, and bias in selection of the reported result. The study will be classified as having a low risk of bias only when all five domains are rated as low risk. For the two *post hoc* analyses derived from CVOTs, the Instrument for assessing the Credibility of Effect Modification Analyses (ICEMAN-RCT) was further utilized to assess the credibility of effect-modification conclusions. The ICEMAN-RCT tool comprises five core items and one optional supplementary item, with the following response options for each core item: definitely yes, probably yes, probably no, definitely no, and not applicable. Overall credibility of each effect-modification analysis is graded as high, moderate, low, or very low credibility according to the synthesis of individual item responses, prior biological–clinical evidence, interaction *p*-values, multiple testing, the definition of the effect modifier, and statistical power. Notably, RoB 2.0 evaluates the methodological bias of the original RCTs, whereas ICEMAN-RCT specifically assesses the credibility of *post hoc* effect modification analyses. A low risk of bias in the original RCT does not necessarily imply that the conclusions of its *post hoc* analysis are credible. All discrepancies during bias assessment were resolved by discussion, with the corresponding author providing arbitration when consensus could not be reached.

### Statistical analyses

Statistical analyses were performed using STATA 14.0, RStudio (version 4.3.3), and GraphPad Prism software. HRs with 95% CIs were used as effect estimates. Heterogeneity between studies was quantified by Cochran’s *Q* test and the *I*^2^ statistic; *I*^2^ < 25% was defined as low heterogeneity, 25% < *I*^2^ < 50% as moderate heterogeneity, and *I*^2^ > 50% as high heterogeneity. Considering the inherent clinical heterogeneity across included studies, we prespecified the random-effects model for primary analyses. Fixed-effects model sensitivity analyses were additionally performed to assess the robustness of findings under different statistical assumptions. All corresponding effect estimates are presented in **Supplementary Table S6**. A node-splitting method was used to evaluate consistency between direct and indirect comparisons; *p* > 0.05 indicated no significant inconsistency within the evidence network. Surface under the cumulative ranking curve (SUCRA) values ranged from 0% to 100% and were used to rank treatment efficacy, where higher values represented a higher probability of being the optimal intervention for a specific endpoint. Meta-regression was performed with drug class, sample size, HF subtype, and median follow-up duration as covariates to explore potential sources of heterogeneity. Sensitivity analyses were implemented using the leave-one-out method to verify the robustness of the results. Egger’s test was used to detect small-sample effects and publication bias. *p* < 0.05 indicated significant publication bias.

## Results

### Study selection process

A total of 16,411 studies were retrieved from MEDLINE, Embase, CENTRAL, and ClinicalTrials.gov. After removal of duplicates, 7,663 unique records remained for title and abstract screening, and 2,013 full-text articles were retrieved. A total of 10 RCTs (26, 30–37) and two *post hoc* analyses of CVOTs (38, 39) were finally included. The selection process is illustrated in **Figure 1**.

**Figure 1:**
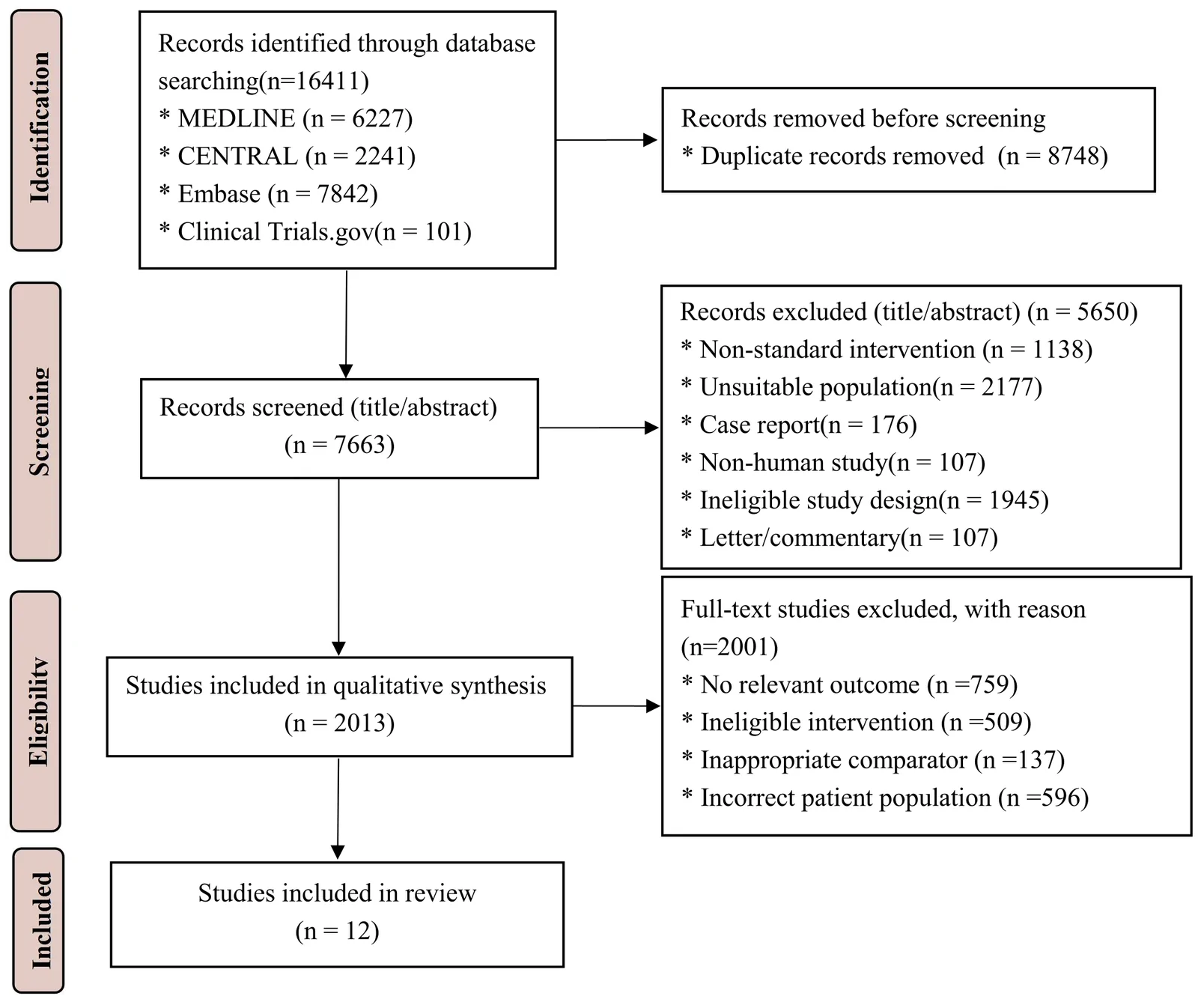
Study selection process.

### Inconsistency testing

No closed loops existed within any of the subgroup evidence networks. There were no intervention pairs that simultaneously had both direct and indirect comparisons, so the criteria for applying node-splitting were not met. Therefore, inconsistency assessment could not be conducted in this study.

### Baseline characteristics of included studies

Baseline characteristics of included studies are summarized in **Table 2** and **Supplementary Table S5**, which also summarizes event counts for each outcome and treatment comparison. The pooled population consisted of 13,886 patients with T2DM and HF; 11,467 of them were included in SGLT-2is studies, 1,794 of them were included in GLP-1RAs studies, 371 of them were included in GIP/GLP-1RAs studies, and 254 of them were included in DPP-4is studies. The included studies comprised 10 RCTs and 2 *post hoc* analyses of CVOTs. The majority of the included studies were two-arm studies and were published between 2018 and 2025. The follow-up duration of these studies ranged from 0.5 to 5.4 years.

**Table 2:** Baseline characteristics for included randomized evidence.

Study	Trials	Registration number	Treatment	Sample size (*n*)	Age (year)	Male sex (*n*)	eGFR (mL/min/1.73 m^2^)	HbA1c (%)	Population (follow-up duration)
Ueda et al. (30)	CANONICAL	UMIN 000028668	Canagliflozin	42	76.5 ± 6.4	55	57.0 ± 14.0	7.01 ± 0.7	T2DM with HFpEF and ≥ 65 years (24 weeks)
			Placebo	40	75.9 ± 5.8				
Branch et al. (39)	REWIND	NCT01394952	Dulaglutide	421	65.7 ± 7.4	488	71.0 ± 21.1	7.3 ± 1.0	T2DM with HF and ASCVD (median: 5.4 years)
			Placebo	432					
Inzucchi et al. (31)	DELIVER	NCT03619213	Dapagliflozin	3,150	71.0 ± 9.1	1,818	59.5 ± 19.5	7.4 ± 1.6	T2DM with HFmrEF and ≥ 40 years (median: 2.3 [IQR: 1.7–2.8] years)
			Placebo						
Pellicori et al. (38)	EMPA-REG OUTCOME	NCT01131671	Empagliflozin	706	64.5 ± 8.8	495	68.7 ± 20.4	NR	T2DM with HF (1 year)
			Placebo						
Filippatos et al. (32)	EMPEROR-Preserved	NCT03057951	Empagliflozin	2,938	70.9 ± 9.0	1,682	59.7 ± 20.7	7.26 ± 1.5	T2DM with HFmrEF (26.2 months)
			Placebo						
Anker et al. (33)	EMPEROR-Reduced	NCT03057977	Empagliflozin	927	66.8 ± 10.0	1,482	61.2 ± 22.2	7.4 ± 1.6	T2DM with HFrEF (median: 16 months)
			Placebo	929	66.6 ± 10.3				
Kosiborod et al. (34)	FLOW	NCT03819153	Semaglutide	167	NR	NR	NR	NR	T2DM with HF (median: 3.5 years)
			Placebo	158	NR				
Kosiborod et al. (34)	STEP-HFpEF DM	NCT04916470	Semaglutide	310	NR	NR	NR	NR	T2DM with HFpEF (57 weeks)
			Placebo	306	NR				
Pitt et al. (40)	SOLOIST-WHF	NCT03521934	Sotagliflozin	290	69 ± 8.9	389	51.2 ± 16.1	7.2 ± 1.3	T2DM with HF (median: 9 months)
			Placebo	306	70 ± 8.9				
Packer et al. (26)	SUMMIT	NCT04847557	Tirzepatide	182	65.3 ± 9.9	186	NR	NR	T2DM with HFpEF (median: 104 weeks)
			Placebo	189	64.9 ± 10.0		NR	NR	
McMurray et al. (36)	VIVIDD	NCT00894868	Vildagliptin	128	62.9 ± 8.5	195	NR	NR	T2DM with HFrEF (52 weeks)
			Placebo	126	63.4 ± 10.2		NR	NR	
Petrie et al. (37)	DAPA-HF	NCT03036124	Dapagliflozin	1,075	66.3 ± 9.9	1,662	NR	NR	T2DM with HFrEF (median: 18 months)
			Placebo	1,064	66.7 ± 9.8				

NR, not reported; HFpEF, heart failure with preserved ejection fraction; HFrEF, heart failure with reduced ejection fraction; HFmrEF, heart failure with mildly reduced ejection fraction; ASCVD, atherosclerotic cardiovascular disease; eGFR, estimated glomerular filtration rate.

### Risk of bias

All original RCTs were deemed to be at low risk of bias overall (**Figure 2**). Credibility assessment showed that both *post hoc* CVOT analyses were graded as low credibility (**Table 3**). Neither analysis explicitly stated prespecified hypotheses for effect modification (definitely no). Nevertheless, existing mechanistic and clinical evidence suggests potential treatment effect modification by baseline HF status (probably yes). Formal interaction tests in both studies yielded not statistically significant results; chance could not be ruled out as an explanation for the observed subgroup point estimates (definitely no). Branch et al. performed multiple *post hoc* outcome analyses without adjustment for multiplicity (definitely no), whereas Pellicori et al., 2020 reported multiple exploratory analyses also without adjustment for multiplicity (probably no). As the effect modifier was a binary variable, the item assessing arbitrary cut-points for continuous variables was rated as “not applicable”. In summary, these two *post hoc* effect modification analyses were rated as providing low-credibility evidence. Findings from these analyses are hypothesis-generating only and cannot serve as confirmatory evidence for differential treatment effects across subgroups defined by baseline HF.

**Figure 2:**
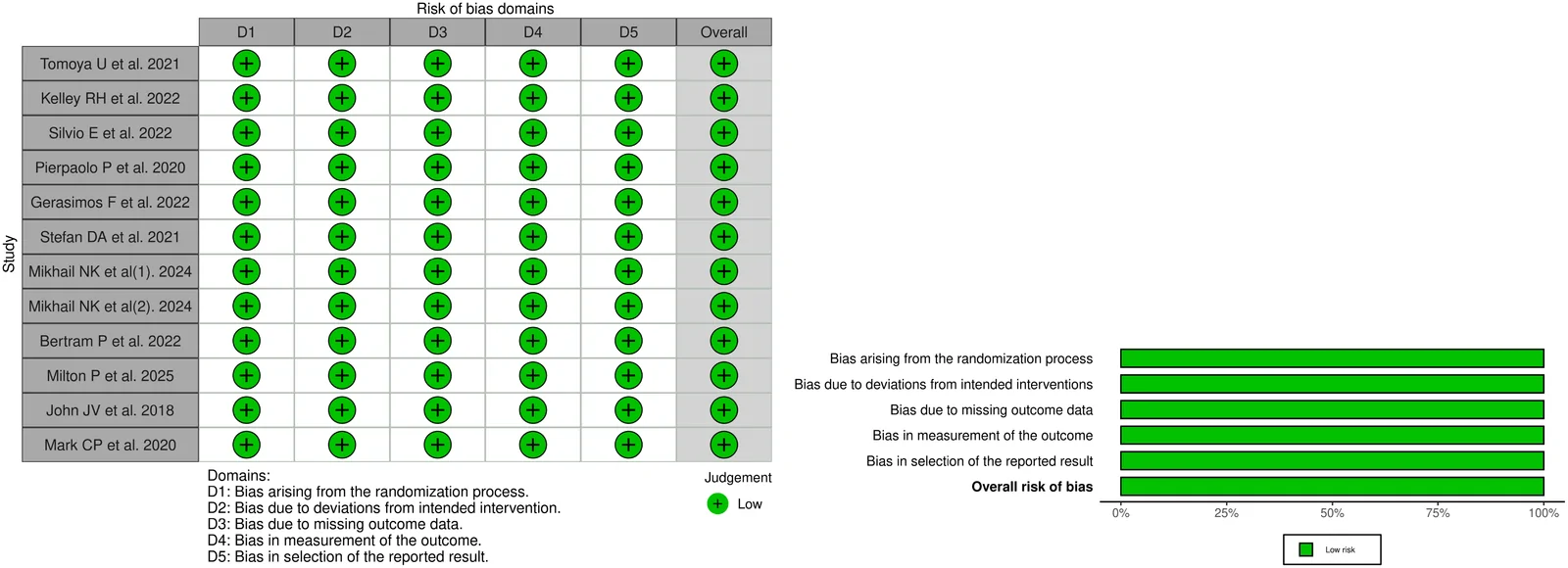
Risk of bias summary of included RCTs and *post hoc* CVOTs.

**Table 3:** Credibility assessment of subgroup analyses by HF grouping.

ICEMAN-RCT item	Branch et al. (39)	Pellicori et al. (38)
Was the direction of effect modification correctly hypothesized *a priori*?	Definitely no	Definitely no
Was the effect modification supported by prior evidence?	Probably yes	Probably yes
Does a test for interaction suggest that chance is an unlikely explanation of the apparent effect modification?	Definitely no	Definitely no
Did the authors test only a small number of effect modifiers or consider the number in their statistical analysis?	Definitely no	Probably no
If the effect modifier is a continuous variable, were arbitrary cut points avoided?	Not applicable	Not applicable
Are there any additional considerations that may increase or decrease credibility?	Not applicable	Not applicable
How would you rate the overall credibility of the proposed effect modification?	Low credibility	Low credibility

### GRADE quality assessment

Regarding the HF-related composite endpoint, there were 11 direct placebo-controlled comparisons across included studies, with one study rated as high quality, one study rated as moderate quality, and nine studies rated as low quality. For worsening of HF, there were eight direct comparisons with placebo, with four studies rated as high quality, one study rated as moderate quality, and three studies rated as low quality. For hospitalization for HF, there were six direct comparisons in the original studies, all of which were rated as low quality. For CV death, there were nine direct comparisons with placebo, with six studies rated as high quality and three studies rated as low quality. In terms of all-cause death, there were six direct comparisons with placebo, with one study rated as high quality, one study rated as moderate quality, and four studies rated as moderate quality. Regarding the renal composite outcome, there were three direct comparisons with placebo in the original studies, all of which were rated as high quality. GRADE quality assessment results are summarized in **Table 4**.

**Table 4:** GRADE assessment.

Certainty assessment							Certainty	Importance
Comparison	No. of studies	Study design	Risk of bias	Inconsistency	Indirectness	Imprecision		
HF-related composite endpoint								
SGLT-2is vs. placebo	6	RCT, *post hoc* analyses of CVOTs	Serious	Unable to assess	Not serious	Serious	⨁⨁◯◯	Critical
							Low
GLP-1RAs vs. placebo	3	RCT, *post hoc* analyses of CVOTs	Serious	Unable to assess	Not serious	Serious	⨁⨁◯◯	Critical
							Low
GIP/GLP-1 vs. placebo	1	RCT	Not serious	Unable to assess	Not serious	Not serious	⨁⨁⨁⨁	Critical
							High
DPP-4is vs. placebo	1	RCT	Not serious	Unable to assess	Not serious	Serious	⨁⨁⨁◯	Critical
							Moderate
Worsening of HF								
SGLT-2is vs. placebo	3	RCT	Not serious	Unable to assess	Not serious	Not serious	⨁⨁⨁⨁	Critical
							High
GLP-1RAs vs. placebo	3	RCT, *post hoc* analyses of CVOTs	Serious	Unable to assess	Not serious	Serious	⨁⨁◯◯	Critical
							Low
GIP/GLP-1 vs. placebo	1	RCT	Not serious	Unable to assess	Not serious	Not serious	⨁⨁⨁⨁	Critical
							High
DPP-4is vs. placebo	1	RCT	Not serious	Unable to assess	Not serious	Serious	⨁⨁⨁◯	Critical
							Moderate
Hospitalization of HF								
SGLT-2is vs. placebo	6	RCT, *post hoc* analyses of CVOTs	Serious	Unable to assess	Not serious	Serious	⨁⨁◯◯	Critical
							Low
CV death								
SGLT-2is vs. placebo	5	RCT	Not serious	Unable to assess	Not serious	Not serious	⨁⨁⨁⨁	Critical
							High
GLP-1RAs vs. placebo	3	RCT, *post hoc* analyses of CVOTs	Serious	Unable to assess	Not serious	Serious	⨁⨁◯◯	Critical
							Low
DPP-4is vs. placebo	1	RCT	Not serious	Unable to assess	Not serious	Not serious	⨁⨁⨁⨁	Critical
							High
All-cause death								
SGLT-2is vs. placebo	3	RCT, *post hoc* analyses of CVOTs	Serious	Unable to assess	Not serious	Serious	⨁⨁◯◯	Critical
							Low
GLP-1RAs vs. placebo	1	*Post hoc* analyses of CVOTs	Serious	Unable to assess	Not serious	Serious	⨁⨁◯◯	Critical
							Low
GIP/GLP-1 vs. placebo	1	RCT	Not serious	Unable to assess	Not serious	Not serious	⨁⨁⨁⨁	Critical
							High
DPP-4is vs. placebo	1	RCT	Not serious	Unable to assess	Not serious	Serious	⨁⨁⨁◯	Critical
							Moderate
Renal composite outcome								
SGLT-2is vs. placebo	3	RCT	Not serious	Unable to assess	Not serious	Not serious	⨁⨁⨁⨁	
							High	Critical

### HF-related composite endpoint

Network meta-analysis was performed for the HF-related composite endpoint. Compared with placebo, SGLT-2is (HR [95% CI]: 0.75 [0.68, 0.82]) and GLP-1RAs (HR [95% CI]: 0.77 [0.61, 0.97]) yielded statistically significant reductions in the risk of the HF-related composite endpoint. GIP/GLP-1RAs showed a numerical trend toward benefit versus placebo, although the difference did not reach statistical significance (HR [95% CI]: 0.64 [0.35, 1.16]). No significant difference was observed between SGLT-2is and GLP-1RAs (HR [95% CI]: 0.98 [0.76, 1.25]) or between DPP-4is and placebo (HR [95% CI]: 1.11 [0.68, 1.80]). Notably, all head-to-head comparisons between active pharmacological agents failed to detect statistically significant differences. The results of the sensitivity analysis of the fixed-effects model are consistent in the direction of effect with the primary random-effects model (**Supplementary Table S6**). There was no significant heterogeneity (*I*^2^ = 5.8%, *p* = 0.3852). The corresponding forest plot and network plot are shown in **Figure 3**.

**Figure 3:**
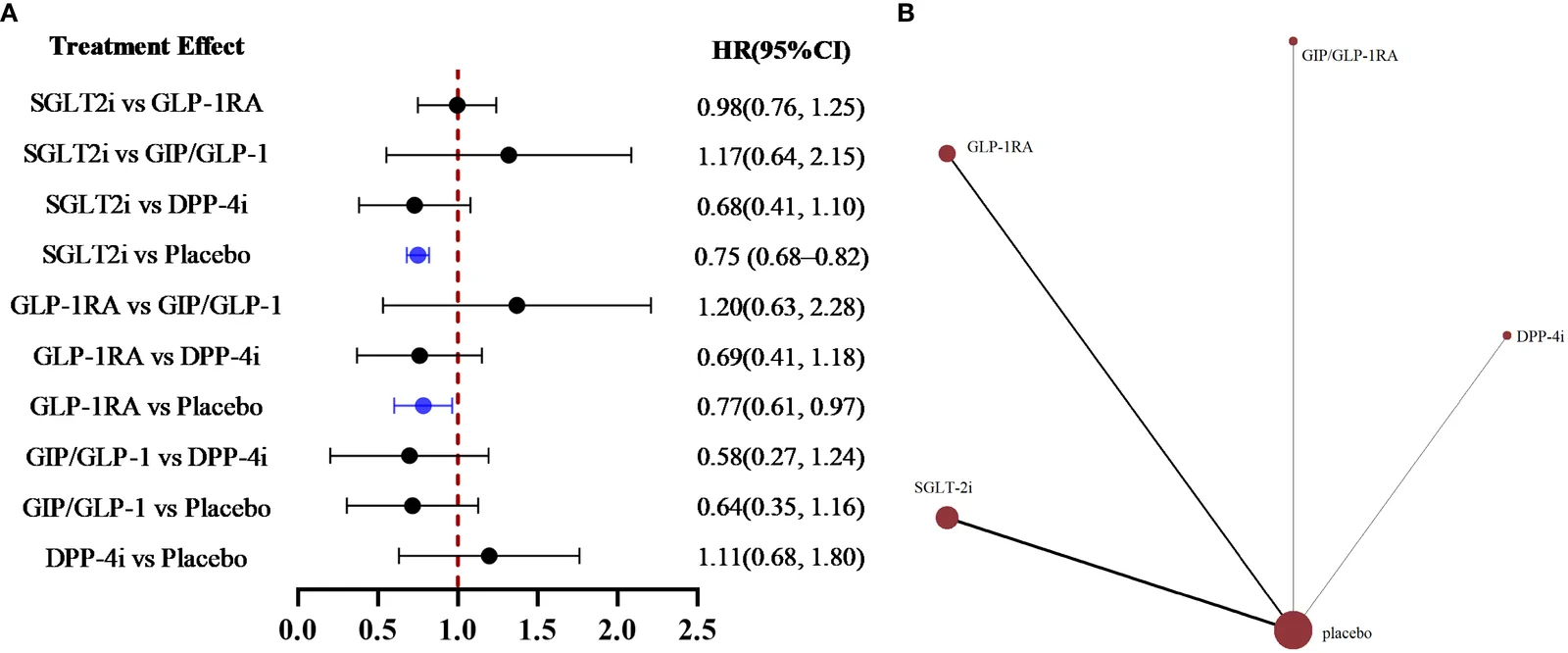
**(A)** Forest plots for network meta-analyses of the HF-related composite endpoint. **(B)** Network plot for the HF-related composite endpoint.

### Worsening of HF

SGLT-2is demonstrated a statistically significant risk reduction for worsening of HF compared with placebo (HR [95% CI]: 0.72 [0.53, 0.96]). No statistically significant differences were observed in all other pairwise comparisons. Under the random-effects model, GLP-1RAs (HR [95% CI]: 0.73 [0.52, 1.04]) and GIP/GLP-1RAs (HR [95% CI]: 0.49 [0.23, 1.03]) showed numerical trends toward reduced risk of HF worsening compared to placebo, while DPP-4is (HR [95% CI]: 1.03 [0.53, 2.01]) exhibited a neutral effect. Of note, GLP-1RAs (HR [95% CI]: 0.75 [0.57, 0.98]) and GIP/GLP-1RAs (HR [95% CI]: 0.49 [0.25, 0.96]) showed nominally significant reductions in the risk of HF worsening compared with placebo under the fixed-effect sensitivity model, indicating that these borderline findings are sensitive to modeling assumptions (**Supplementary Table S6**). There was moderate heterogeneity (*I*^2^ = 29.6%, *p* = 0.2240). The corresponding forest plot and network plot are shown in **Figure 4**.

**Figure 4:**
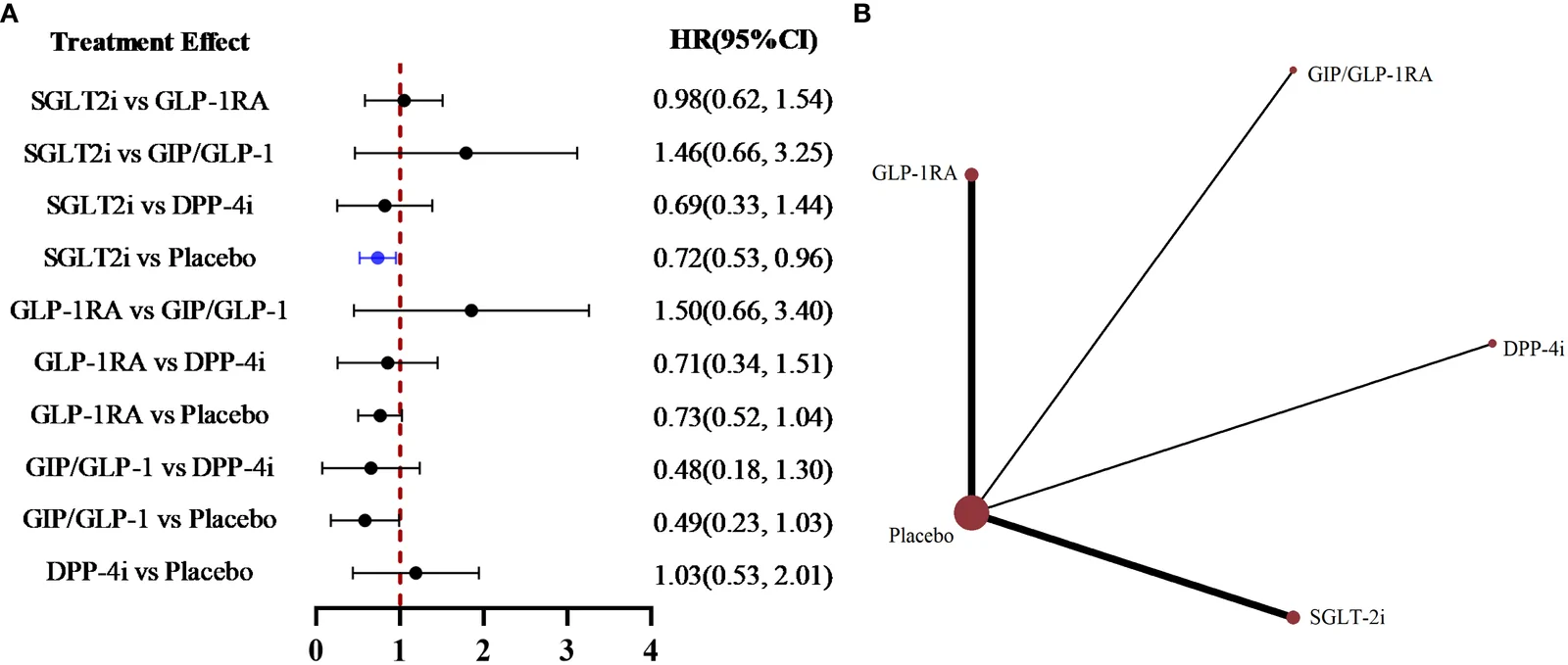
**(A)** Forest plots for network meta-analyses of worsening of HF. **(B)** Network plot for worsening of HF.

### CV death

Compared with placebo, SGLT-2is significantly reduced the risk of CV death (HR [95% CI]: 0.87 [0.77, 0.98]). However, no statistically significant differences were observed for GLP-1RAs vs. placebo (HR [95% CI]: 0.85 [0.62, 1.17]) or DPP-4is vs. placebo (HR [95% CI]: 1.72 [0.50, 5.09]) vs. placebo. Direct comparisons between different active drugs also showed no statistically significant differences in CV death between SGLT-2is and GLP-1RAs (HR [95% CI]: 1.03 [0.73, 1.45]), SGLT-2is and DPP-4is (HR [95% CI]: 0.51 [0.15, 1.75]), or GLP-1RAs and DPP-4is (HR [95% CI]: 0.49 [0.14, 1.76]). The results of the fixed-effect sensitivity analysis were consistent with the direction of effect in the random-effects model (**Supplementary Table S6**). No significant heterogeneity was detected (*I*^2^ = 0.0%, *p* = 0.7165). Detailed results are presented in **Figure 5**.

**Figure 5:**
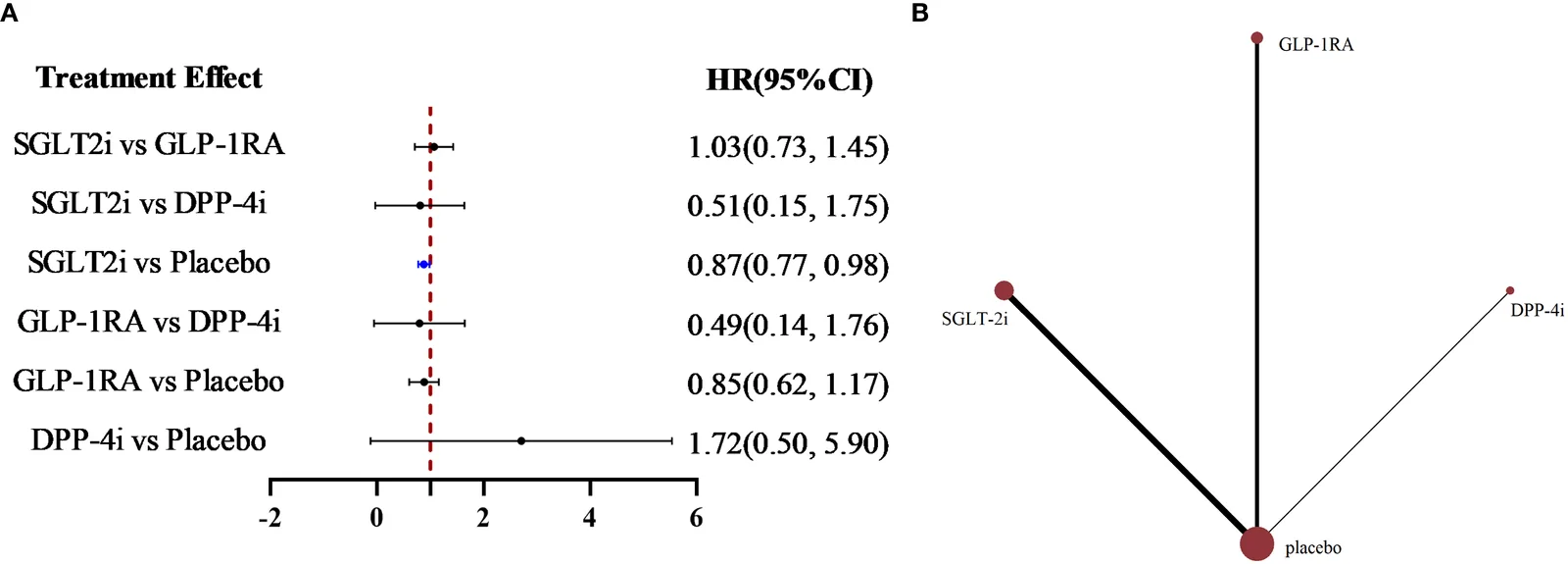
**(A)** Forest plots for network meta-analyses of CV death. **(B)** Network plot for CV death.

### All-cause death

For all-cause death, no pairwise comparisons reached statistical significance under the random-effects model. Compared to placebo, both SGLT-2is (HR [95% CI]: 0.83 [0.67, 1.02]) and GLP-1RAs (HR [95% CI]: 0.92 [0.59, 1.44]) demonstrated nonsignificant numerical trends toward a reduced risk of all-cause death. Of note, the comparison of SGLT-2is vs. placebo attained nominal statistical significance in the fixed-effect sensitivity analysis (HR [95% CI]: 0.89 [0.81, 0.99]), representing a borderline finding susceptible to modeling assumptions. In contrast, GIP/GLP-1RAs showed a nonsignificant numerical trend toward increased risk (HR [95% CI]: 1.50 [0.60, 3.76]). Head-to-head comparisons between active drugs also did not reveal significant differences. In summary, the results of this network meta-analysis indicate that the four classes of novel hypoglycemic agents have not demonstrated significant benefits in reducing all-cause mortality risk in patients with T2DM and HF. There was high between-study heterogeneity (*I*^2^ = 65.0%, *p* = 0.0223). The corresponding forest plot and network plot are shown in **Figure 6**.

**Figure 6:**
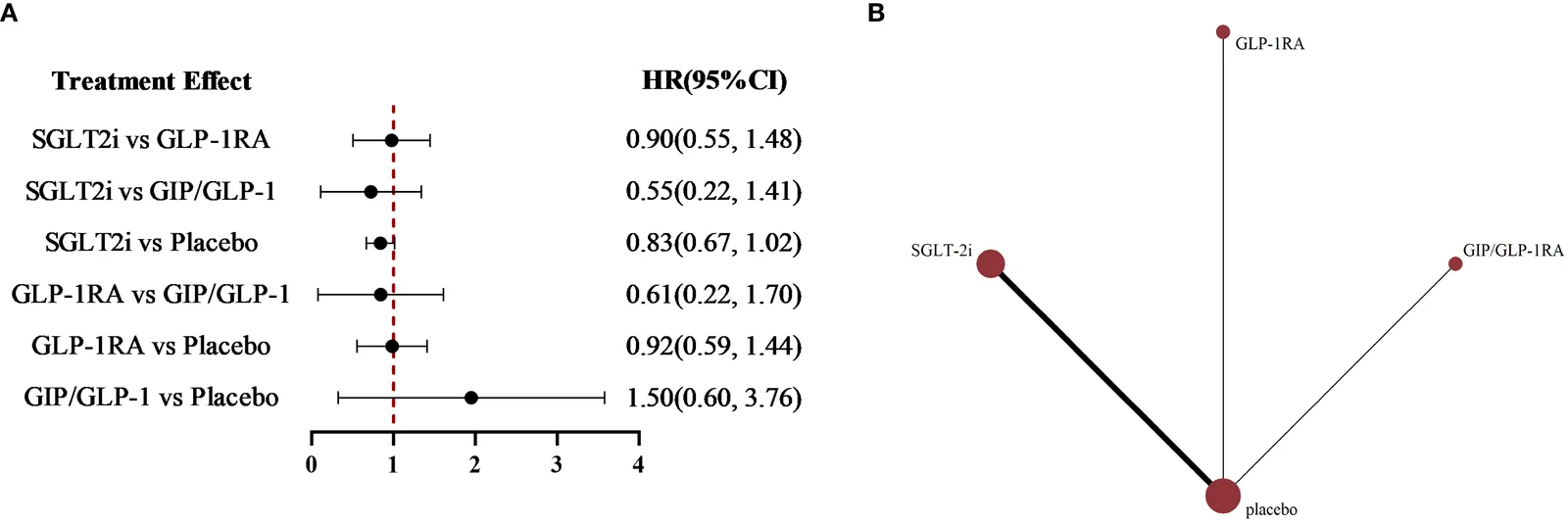
**(A)** Forest plots for network meta-analyses of all-cause death. **(B)** Network plot for all-cause death.

### Hospitalization of HF

This study employed a paired meta-analysis approach to evaluate the effect of SGLT-2is on hospitalization for HF. Six eligible trials were included. The pooled estimates demonstrated that SGLT-2is significantly reduced the risk of hospitalization for HF (HR [95% CI]: 0.74 [0.66, 0.83]). The results of the fixed-effects model showed a consistent direction of effect with the primary random-effects model (**Supplementary Table S6**). There was no heterogeneity between studies (*I*^2^ = 0.0%, *p* = 0.645). Detailed results are shown in **Figure 7A**.

**Figure 7:**
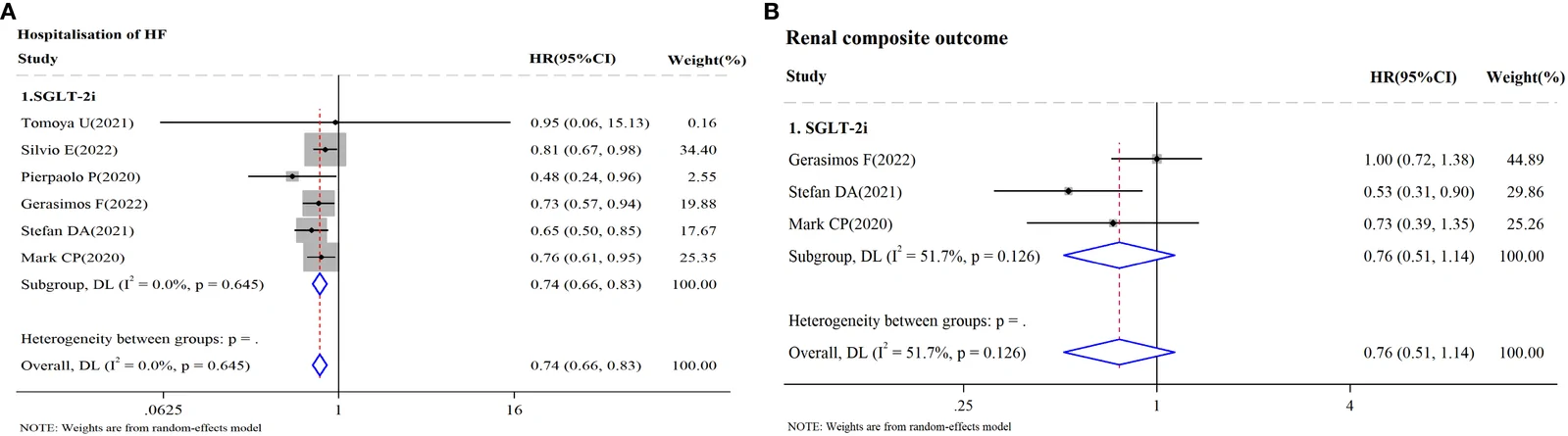
**(A)** Forest plot for hospitalization for HF. **(B)** Forest plot for renal composite outcome.

### Renal composite outcome

To evaluate the renal effects of SGLT-2is, a paired meta-analysis of renal composite outcomes was conducted. 3 trials were included. There was moderate heterogeneity between studies (*I*^2^ = 51.7%, *p* = 0.126). The pooled point estimate indicated a numerical trend toward renal protection with SGLT-2is. However, the results did not reach statistical significance [HR with 95%CI: 0.76(0.51, 1.14)]. Individual trial results were inconsistent: 1 trial showed that SGLT-2is significantly reduced the incidence of the renal composite event [HR with 95%CI: 0.53(0.31, 0.90)], while the other two studies demonstrated no significant difference, including the largest-weighted trial, which also failed to observe a treatment effect [HR with 95%CI: 1.00(0.72, 1.38), HR with 95%CI: 0.73(0.39, 1.35)]. And the results of the fixed-effects model showed consistent effect directions relative to the primary random-effects model (**Supplementary Table 6**). Detailed results are presented in **Figure 7B**.

### Sensitivity analysis

This study evaluated potential publication bias using Egger’s test and assessed the robustness of the pooled effect through leave-one-out sensitivity analysis. Egger’s test indicated no significant publication bias (*p* = 0.253) (**Supplementary Figure S1**: Egger’s test). The leave-one-out sensitivity analysis revealed that upon sequential exclusion of each study, the pooled risk ratio exhibited minor fluctuations, and the direction of the pooled effect size remained consistent with the original meta-analysis (**Supplementary Figure S2**: Leave-one-out sensitivity analysis). In summary, this meta-analysis showed no evidence of significant publication bias, and the overall research findings are robust and reliable. Considering clinical heterogeneity among the included studies in terms of baseline characteristics, follow-up duration, and drug dosages, this study conducted a sensitivity analysis using a fixed-effects model to evaluate the robustness of the pooled results under different statistical assumptions. The pooled effect sizes from both models are detailed in **Supplementary Table S6** (Summary of effect estimates for all outcomes under random-effects versus fixed-effects models). The results indicated that the HRs and directions of effect for the majority of outcome measures were largely consistent between the random-effects and fixed-effects models. Only minor changes in CIs were observed for two comparisons of HF deterioration (GLP-1RAs vs. placebo, GIP/GLP-1s vs. placebo): the effects were statistically significant under the fixed-effects model but not under the random-effects model, suggesting that the results of these two comparisons may be sensitive to model selection.

Since sotagliflozin is a dual SGLT1/SGLT2i, its pharmacological mechanism differs partially from that of selective SGLT2is. Therefore, we conducted a sensitivity analysis excluding the Effect of Sotagliflozin on Cardiovascular Events in Patients With Type 2 Diabetes Post Worsening Heart Failure (SOLOIST-WHF) trial to evaluate the impact of classifying sotagliflozin as an SGLT2i (as shown in **Supplementary Table S7**: Summary of effect estimates after excluding the SOLOIST-WHF in random-effects models). The effect estimates and statistical conclusions for the HF-related composite endpoint, CV death, and all-cause death remained robust. Regarding worsening of HF, after excluding SOLOIST-WHF, the comparisons of GLP-1RAs versus placebo and GIP/GLP-1RA vs. placebo shifted from not statistically significant (GLP-1RAs vs. placebo: 0.73 [0.52, 1.04], GIP/GLP-1RA vs. placebo: 0.49 [0.23, 1.03]) to statistically significant (GLP-1RAs vs. placebo: 0.75 [0.57, 0.98], GIP/GLP-1RA vs. placebo: 0.49 [0.25, 0.96]). The point estimates of HRs changed minimally, with the primary change arising from the contraction of 95% CIs. This suggests that these two indirect comparisons are statistically borderline and sensitive to the exclusion of SOLOIST-WHF, and the results should be interpreted with caution.

### Subgroup analyses and meta-regression

Low between-study heterogeneity was observed for the HF-related composite endpoint (*I*^2^ = 5.8%) and CV death (*I*^2^ = 0.0%); therefore, prespecified subgroup analyses and meta-regression were not performed for these outcomes. For worsening of HF, moderate between-study heterogeneity was observed (*I*^2^ = 29.6%). Predefined subgroup analyses stratified by HF subtype and drug class were conducted, revealing variable residual heterogeneity within each stratum (HF: *I*^2^ = 69.1%; HFpEF: *I*^2^ = 54.5%; SGLT-2is: *I*^2^ = 42.2%; GLP-1RAs: *I*^2^ = 10.0%). Univariate meta-regression demonstrated that follow-up duration (*p* = 0.48), HF subtype (*p* = 0.66), and drug class (*p* = 0.57) could not independently explain the observed between-study heterogeneity. Multivariate meta-regression was subsequently performed to adjust for covariates, and the global joint test for all included covariates failed to reach statistical significance (*p* = 0.089). For all-cause death, high between-study heterogeneity was observed (*I*^2^ = 68.1%, *p* = 0.02). Univariate meta-regression results indicated that follow-up duration (*p* = 0.41), HF subtype (*p* = 0.62), drug class (*p* = 0.75), and sample size (*p* = 0.25) could not independently explain between-study differences in effect estimates. Multivariable meta-regression also showed no overall statistically significant association for the covariates (*p* = 0.52). This suggests that based on the currently included studies, there is insufficient evidence to identify follow-up duration, HF subtype, drug class, or sample size as effect modifiers for the impact of novel hypoglycemic agents on all-cause death. Limited by the number of included studies, the foregoing meta-regression analysis should be considered exploratory.

## Discussion

T2DM and HF share overlapping pathophysiological mechanisms that mediate the progression of both diseases and form a vicious cycle. Chronic hyperglycemia induces oxidative stress, systemic inflammation, and endothelial dysfunction, all of which promote the progression of microvascular and macrovascular complications, thereby increasing the risk of HF (41). Long-term hyperglycemia can also induce myocardial hypertrophy and interstitial fibrosis, leading to diabetic cardiomyopathy (42). Furthermore, cardiac dysfunction exacerbates insulin resistance and impairs glycemic control, ultimately resulting in an adverse long-term prognosis (41). This study focuses on patients with T2DM complicated by HF and categorizes GIP/GLP-1RAs separately. Through network meta-analysis, it systematically compares the efficacy of four classes of novel hypoglycemic agents in terms of HF-related composite endpoints and renal composite endpoints. The results showed that SGLT-2is and GLP-1RAs both exhibit distinct cardiovascular benefits, while DPP-4is did not demonstrate meaningful cardiac protection and showed a trend toward increased risks of CV death and all-cause death. This study provides high-quality evidence to support glycemic management for patients with T2DM and HF.

SGLT-2is such as dapagliflozin and empagliflozin promote urinary glucose excretion by inhibiting proximal tubular glucose reabsorption, thereby exerting glucose-lowering and weight-reducing effects and inducing osmotic diuresis independently of insulin. Their cardiorenal protective effects are partially mediated by natriuretic and diuretic actions, which reduce vascular volume load, lower cardiac preload and afterload, and improve hemodynamic status (43). Beyond volume regulation, SGLT-2is can also maintain myocardial homeostasis through multiple pathways, including optimizing myocardial energy metabolism, inhibiting myocardial fibrosis, and exerting anti-inflammatory and antioxidant effects, collectively forming a comprehensive cardiorenal protection network (44, 45). Consistent with the results of this network meta-analysis, multiple large CVOTs have confirmed that the aforementioned multitarget mechanisms can sustainably reduce the risk of hospitalization for HF and cardiovascular mortality (31, 38, 43), with significant benefits for HF-related outcomes regardless of T2DM status (46). This network meta-analysis further confirms that SGLT-2is demonstrate significant benefits in HF-related composite endpoints, HF hospitalization, HF worsening, and CV death. However, no statistically significant benefits were observed for all-cause death and renal composite endpoints.

Sotagliflozin is the first approved SGLT-1/SGLT-2 dual inhibitor, with a selectivity for SGLT-2 approximately 20 times that of SGLT-1, offering dual regulatory advantages in the kidneys and intestines. Inhibiting renal SGLT-2 promotes urinary glucose excretion, while inhibiting intestinal SGLT-1 reduces glucose absorption and lowers postprandial blood glucose (47). Its unique dual-target mechanism has positioned it as a novel therapeutic agent for HF. The SOLOIST-WHF trial demonstrated that initiating sotagliflozin treatment stabilization during hospitalization or early after discharge in T2DM patients complicated by recent HF decompensation significantly reduced the composite risk of CV death, HF hospitalization, and HF emergency department visits (48). The SCORED trial, which focused on cardiovascular high-risk individuals with T2DM and chronic kidney disease, similarly confirmed that sotagliflozin significantly reduced the composite risk of HF-related events and exhibited potential protective effects against myocardial infarction and stroke (49). Incorporation of sotagliflozin-related data into the present network meta-analysis did not result in significant changes to the overall core conclusions, supporting the robustness of the cardioprotective effects of SGLT-2is. Currently, the specific mechanisms underlying the cardiovascular benefits of sotagliflozin are not fully elucidated, and there is a lack of head-to-head clinical trials confirming whether its dual-target inhibition provides additional clinical benefits compared with traditional selective SGLT-2is. Overall, SGLT-2is consistently demonstrate a trend toward improved cardiovascular outcomes. Differences in efficacy among different drugs may arise from heterogeneity in clinical trials and subtle differences in the comorbidity profiles of the study populations. Future high-quality research is still needed to further explore and validate the conclusions of this study (50).

GLP-1RAs improve glycemia, body weight, and insulin resistance by activating systemic GLP-1 receptors (51), and their cardiorenal benefits are independent of metabolic improvements (15). Basic research indicates that the cardiovascular protective effects of these drugs are not limited to lipid modulation and weight reduction. They can directly inhibit vascular inflammation, delay atherosclerotic progression, and promote repair of ischemic myocardial injury, thereby improving long-term cardiovascular outcomes (51, 52). Clinical evidence further suggests that semaglutide may produce early cardiovascular benefits prior to significant weight loss. The SELECT trial also confirmed no significant association between weight loss magnitude and the reduction in the risk of cardiovascular adverse events (53). The renal protective mechanisms of GLP-1RAs remain controversial. In animal models, GLP-1 receptor signaling can bidirectionally regulate renal function. In humans, however, GLP-1 receptors in the kidneys are primarily expressed in vascular smooth muscle, with extremely low expression levels in the glomerulus and renal tubules. Current consensus holds that systemic anti-inflammation is the core pathway through which GLP-1RAs mediate cardiorenal protection (54, 55). Integration of mechanistic studies suggests that SGLT2is and GLP-1RAs exhibit cardiorenal protective effects independent of glycemic control and possess complementary features (56, 57). SGLT2is primarily reduce cardiac load through hemodynamic improvement and regulate myocardial metabolism through epicardial adipose tissue (58, 59). By contrast, GLP-1RAs primarily exert antiatherosclerotic and systemic anti-inflammatory effects (58, 60, 61). Combination therapy with the two drug classes may achieve synergistic cardiovascular protection, but further clinical trials are needed to validate this (60).

GIP/GLP-1RAs are a novel class of incretin-based drugs that combine the anti-inflammatory and antiatherosclerotic properties of GLP-1 single-target agonists with the activation of GIP receptors to remodel myocardial energy metabolism and stabilize volume homeostasis (62). Theoretically, the synergistic effect of the dual targets is expected to slow the progression of HF and reduce acute HF-related adverse events (63–65). Representative agents such as tirzepatide demonstrate significant glucose-lowering and weight-reducing effects, which can alleviate cardiac mechanical load (63). The SUMMIT trial confirmed that tirzepatide improves HF-related symptoms and biomarkers, reducing the risk of cardiovascular death and HF hospitalization (26). The SURPASS-2 trial also showed that its cardiovascular outcomes are superior to those of conventional diabetes treatment regimens (66). Even in nondiabetic populations, tirzepatide still reduces the risk of HF, major adverse cardiovascular events, and chronic kidney disease (67). However, in this study, which included patients with T2DM and HF, GIP/GLP-1RAs did not show statistically significant benefits in HF-related composite endpoints and renal composite endpoints. This may be related to the limited number of included trials, small sample sizes, and insufficient follow-up durations. Future large-scale, long-term clinical trials and real-world cohort studies are still needed to further validate their sustained cardiorenal protective effects in patients with T2DM complicated by HF.

DPP-4is exert glucose-lowering effects by inhibiting DPP-4 activity and reducing the degradation of endogenous incretins. However, these agents do not possess diuretic effects, myocardial metabolic regulation, antifibrotic properties, or broad-spectrum anti-inflammatory activity. Consequently, they are unable to intervene in the core pathological mechanisms of HF and do not exhibit direct cardiovascular protective effects (68). The results of this study indicate that DPP-4is did not demonstrate protective effects across all included cardiorenal endpoints. Instead, a trend toward increased risk of HF-related composite endpoints, cardiovascular death, and worsening HF was observed. This finding is consistent with the conclusion from classical CVOTs regarding the heterogeneity among DPP-4is in their association with HF risk. The SAVOR-TIMI 53 trial suggested that saxagliptin increased the risk of HF hospitalization in patients with T2DM and HF (69). In the TECOS trial, sitagliptin exhibited neutral effects on cardiovascular endpoints (70, 71), and the EXAMINE trial observed a trend toward increased HF risk with alogliptin, although this difference was not statistically significant (72). Collectively, these findings indicate that the risk of HF-related adverse events is not a uniform class effect of DPP-4is but rather exhibits specificity related to drug subtypes and target populations. In light of these results, there is currently no evidence to support the use of DPP-4is for cardiorenal benefits in patients with T2DM and HF. Their clinical priority is therefore limited. Furthermore, these findings underscore the necessity of mandatory cardiovascular outcome assessments for all novel hypoglycemic agents.

In summary, the results of this study suggest that SGLT-2is and GLP-1RAs can improve multiple cardiorenal endpoints in T2DM patients with HF, demonstrating considerable clinical benefits. In contrast, whether GIP/GLP-1RAs and DPP-4is can provide long-term cardiorenal benefits remains to be validated in further clinical studies. The above conclusions provide evidence-based support for the clinical selection of various new antidiabetic drugs and provide a basis for formulating individualized glycemic control strategies based on patients’ risk profiles and treatment goals.

## Limitations

This study also has certain limitations. First, there is heterogeneity in the definitions of renal outcome endpoints across included studies, as the components and criteria for determining these endpoints are not entirely consistent across studies, which may introduce measurement bias. Second, existing research evidence for GIP/GLP-1RAs and DPP-4is is relatively limited, with a lack of long-term hard endpoint data to support their sustained cardiorenal protective effects. Third, due to restrictions on publicly available raw data, this study was unable to conduct stratified subgroup analyses based on key factors such as baseline renal function and age, making it difficult to provide precise individualized medication recommendations for different high-risk subgroups. In summary, the conclusions of this study should be interpreted cautiously in light of the inherent methodological limitations (73). Future large-scale, head-to-head cardiovascular outcome RCTs are needed to further validate the long-term efficacy and safety of various novel hypoglycemic agents in patients with T2DM complicated by HF, thereby providing higher-level evidence to support precision treatment.

## Conclusion

Optimal clinical management of T2DM requires a comprehensive assessment of cardiovascular and renal comorbidities. This network meta-analysis indicates that SGLT-2is significantly reduce the risk of HF-related composite endpoints and CV death in T2DM patients with HF, although their renoprotective effect does not reach statistical significance. GLP-1RAs also demonstrate beneficial effects on HF-related composite endpoints. These findings suggest that both SGLT-2is and GLP-1RAs are preferred glycemic control strategies for improving cardiovascular outcomes in T2DM patients with HF, while further evidence is needed to confirm their definitive renal benefits.

## Data Availability

The original contributions presented in the study are included in the article/**Supplementary Material**. Further inquiries can be directed to the corresponding author.

## References

[1] Genitsaridi I Salpea P Salim A Sajjadi SF Tomic D James S . 11th edition of the IDF Diabetes Atlas: global, regional, and national diabetes prevalence estimates for 2024 and projections for 2050. Lancet Diabetes Endocrinol. (2026) 14:149–56. doi: 10.1016/s2213-8587(25)00299-2 41412135

[2] Mosenzon O Alguwaihes A Leon JLA Bayram F Darmon P Davis TME . CAPTURE: a multinational, cross-sectional study of cardiovascular disease prevalence in adults with type 2 diabetes across 13 countries. Cardiovasc Diabetol. (2021) 20:154. doi: 10.1186/s12933-021-01344-0 34315481 PMC8317423

[3] Pop-Busui R Januzzi JL Bruemmer D Butalia S Green JB Horton WB . Heart failure: an underappreciated complication of diabetes. A consensus report of the American Diabetes Association. Diabetes Care. (2022) 45:1670–90. doi: 10.2337/dci22-0014 35796765 PMC9726978

[4] Dei Cas A Khan SS Butler J Mentz RJ Bonow RO Avogaro A . Impact of diabetes on epidemiology, treatment, and outcomes of patients with heart failure. JACC Heart Fail. (2015) 3:136–45. doi: 10.1016/j.jchf.2014.08.004 25660838

[5] Khan M Hashim MJ King JK Govender RD Mustafa H Al KJ . Epidemiology of type 2 diabetes - global burden of disease and forecasted trends. J Epidemiol Glob Health. (2020) 10:107–11. doi: 10.2991/jegh.k.191028.001 PMC7310804 32175717

[6] Panchal K Lawson C Chandramouli C Lam C Khunti K Zaccardi F . Diabetes and risk of heart failure in people with and without cardiovascular disease: systematic review and meta-analysis. Diabetes Res Clin Pract. (2024) 207:111054. doi: 10.1016/j.diabres.2023.111054 38104900

[7] Zareini B Blanche P D'Souza M Elmegaard Malik M Nørgaard CH Selmer C . Type 2 diabetes mellitus and impact of heart failure on prognosis compared to other cardiovascular diseases: a nationwide study. Circ Cardiovasc Qual Outcomes. (2020) 13:e6260. doi: 10.1161/circoutcomes.119.006260 32571092

[8] Bannuru RR . Introduction and methodology: standards of care in overweight and obesity-2025. BMJ Open Diabetes Res Care. (2025) 13(Suppl 1):e004928. doi: 10.1136/bmjdrc-2025-004928 40379435 PMC13059890

[9] Kalyani RR Neumiller JJ Maruthur NM Wexler DJ . Diagnosis and treatment of type 2 diabetes in adults: a review. JAMA. (2025) 334:984–1002. doi: 10.1001/jama.2025.5956 40549398 PMC13014273

[10] Lee MMY Sattar N Pop-Busui R Deanfield J Emerson SS Inzucchi SE . Cardiovascular and kidney outcomes and mortality with long-acting injectable and oral glucagon-like peptide 1 receptor agonists in individuals with type 2 diabetes: a systematic review and meta-analysis of randomized trials. Diabetes Care. (2025) 48:846–59. doi: 10.2337/dc25-0241 40156846

[11] Zannad F Ferreira JP Pocock SJ Anker SD Butler J Filippatos G . SGLT2 inhibitors in patients with heart failure with reduced ejection fraction: a meta-analysis of the EMPEROR-Reduced and DAPA-HF trials. Lancet. (2020) 396:819–29. doi: 10.1016/s0140-6736(20)31824-9 32877652

[12] Kristensen SL Rørth R Jhund PS Docherty KF Sattar N Preiss D . Cardiovascular, mortality, and kidney outcomes with GLP-1 receptor agonists in patients with type 2 diabetes: a systematic review and meta-analysis of cardiovascular outcome trials. Lancet Diabetes Endocrinol. (2019) 7:776–85. doi: 10.1016/s2213-8587(19)30249-9 31422062

[13] Correction to: 2023 ESC Guidelines for the management of cardiovascular disease in patients with diabetes: developed by the task force on the management of cardiovascular disease in patients with diabetes of the European Society of Cardiology (ESC). Eur Heart J. (2024) 45:518. doi: 10.1093/eurheartj/ehad774 38247449

[14] Pop-Busui R Rasmussen S Deanfield JE Buse JB Marx N Mulvagh SL . Oral semaglutide and heart failure outcomes in persons with type 2 diabetes: a secondary analysis of the SOUL randomized clinical trial. JAMA Intern Med. (2026) 186:426–36. doi: 10.1001/jamainternmed.2025.7774 41627802 PMC12865694

[15] Jorsal A Kistorp C Holmager P Tougaard RS Nielsen R Hänselmann A . Effect of liraglutide, a glucagon-like peptide-1 analogue, on left ventricular function in stable chronic heart failure patients with and without diabetes (LIVE)-a multicentre, double-blind, randomised, placebo-controlled trial. Eur J Heart Fail. (2017) 19:69–77. doi: 10.1002/ejhf.657 27790809

[16] Margulies KB Hernandez AF Redfield MM Givertz MM Oliveira GH Cole R . Effects of liraglutide on clinical stability among patients with advanced heart failure and reduced ejection fraction: a randomized clinical trial. JAMA. (2016) 316:500–8. doi: 10.1001/jama.2016.10260 27483064 PMC5021525

[17] Munaf M Pellicori P Allgar V Wong K . A meta-analysis of the therapeutic effects of glucagon-like peptide-1 agonist in heart failure. Int J Pept. (2012) 2012:249827. doi: 10.1155/2012/249827 22811735 PMC3395195

[18] Xu Y Huang T Zhang Y Ji D Tuttle KR Carrero JJ . Risk of heart failure hospitalization for GLP-1 receptor agonists versus DPP-4 inhibitors or SGLT-2 inhibitors in patients with type 2 diabetes: a target trial emulation. Circulation. (2026) 153:1086–100. doi: 10.1161/circulationaha.125.075157 41732861

[19] Aoyama M Kawase H Bando YK Monji A Murohara T . Dipeptidyl peptidase 4 inhibition alleviates shortage of circulating glucagon-like peptide-1 in heart failure and mitigates myocardial remodeling and apoptosis via the exchange protein directly activated by cyclic AMP 1/Ras-related protein 1 axis. Circ Heart Fail. (2016) 9:e2081. doi: 10.1161/circheartfailure.115.002081 26721911

[20] Okabe K Matsushima S Ikeda S Ikeda M Ishikita A Tadokoro T . DPP (dipeptidyl peptidase)-4 inhibitor attenuates Ang II (angiotensin II)-induced cardiac hypertrophy via GLP (glucagon-like peptide)-1-dependent suppression of Nox (nicotinamide adenine dinucleotide phosphate oxidase) 4-HDAC (histone deacetylase) 4 pathway. Hypertension. (2020) 75:991–1001. doi: 10.1161/hypertensionaha.119.14400 32160098

[21] Takahashi A Asakura M Ito S Min KD Shindo K Yan Y . Dipeptidyl-peptidase IV inhibition improves pathophysiology of heart failure and increases survival rate in pressure-overloaded mice. Am J Physiol Heart Circ Physiol. (2013) 304:H1361–9. doi: 10.1152/ajpheart.00454.2012 23504176

[22] Li L Li S Deng K Liu J Vandvik PO Zhao P . Dipeptidyl peptidase-4 inhibitors and risk of heart failure in type 2 diabetes: systematic review and meta-analysis of randomised and observational studies. BMJ. (2016) 352:i610. doi: 10.1136/bmj.i610 26888822 PMC4772781

[23] Gantz I Chen M Suryawanshi S Ntabadde C Shah S O'Neill EA . A randomized, placebo-controlled study of the cardiovascular safety of the once-weekly DPP-4 inhibitor omarigliptin in patients with type 2 diabetes mellitus. Cardiovasc Diabetol. (2017) 16:112. doi: 10.1186/s12933-017-0593-8 28893244 PMC5594521

[24] Scirica BM Bhatt DL Braunwald E Steg PG Davidson J Hirshberg B . Saxagliptin and cardiovascular outcomes in patients with type 2 diabetes mellitus. N Engl J Med. (2013) 369:1317–26. doi: 10.1056/nejmoa1307684 23992601

[25] Zannad F Cannon CP Cushman WC Bakris GL Menon V Perez AT . Heart failure and mortality outcomes in patients with type 2 diabetes taking alogliptin versus placebo in EXAMINE: a multicentre, randomised, double-blind trial. Lancet. (2015) 385:2067–76. doi: 10.1016/s0140-6736(14)62225-x 25765696

[26] Packer M Zile MR Kramer CM DiMaria JM Baum SJ Litwin SE . Influence of type 2 diabetes on the effects of tirzepatide in patients with heart failure and a preserved ejection fraction with obesity: a prespecified stratification-based analysis. J Am Coll Cardiol. (2025) 86:696–707. doi: 10.1016/j.jacc.2025.06.058 40903131

[27] Packer M Zile MR Kramer CM Baum SJ Litwin SE Menon V . Tirzepatide for heart failure with preserved ejection fraction and obesity. N Engl J Med. (2025) 392:427–37. doi: 10.1056/nejmoa2410027 39555826

[28] Nauck MA D'Alessio DA . Tirzepatide, a dual GIP/GLP-1 receptor co-agonist for the treatment of type 2 diabetes with unmatched effectiveness regrading glycaemic control and body weight reduction. Cardiovasc Diabetol. (2022) 21:169. doi: 10.1186/s12933-022-01604-7 36050763 PMC9438179

[29] Page MJ McKenzie JE Bossuyt PM Boutron I Hoffmann TC Mulrow CD . The PRISMA 2020 statement: an updated guideline for reporting systematic reviews. Syst Rev. (2021) 10:89. doi: 10.1371/journal.pmed.1003583 33781348 PMC8008539

[30] Ueda T Kasama S Yamamoto M Nakano T Ueshima K Morikawa Y . Effect of the sodium-glucose cotransporter 2 inhibitor canagliflozin for heart failure with preserved ejection fraction in patients with type 2 diabetes. Circ Rep. (2021) 3:440–8. doi: 10.1253/circrep.cr-21-0030 34414333 PMC8338435

[31] Inzucchi SE Claggett BL Vaduganathan M Desai AS Jhund PS de Boer RA . Efficacy and safety of dapagliflozin in patients with heart failure with mildly reduced or preserved ejection fraction by baseline glycaemic status (DELIVER): a subgroup analysis from an international, multicentre, double-blind, randomised, placebo-controlled trial. Lancet Diabetes Endocrinol. (2022) 10:869–81. doi: 10.1016/s2213-8587(22)00308-4 36372069

[32] Filippatos G Butler J Farmakis D Zannad F Ofstad AP Ferreira JP . Empagliflozin for heart failure with preserved left ventricular ejection fraction with and without diabetes. (2022) 146:676–86. doi: 10.1161/circulationaha.122.059785 PMC9422757 35762322

[33] Anker SD Butler J Filippatos G Khan MS Marx N Lam CSP . Effect of empagliflozin on cardiovascular and renal outcomes in patients with heart failure by baseline diabetes status: results from the EMPEROR-Reduced trial. (2021) 143:337–49. doi: 10.1161/circulationaha.120.051824 PMC7834911 33175585

[34] Kosiborod MN Deanfield J Pratley R Borlaug BA Butler J Davies MJ . Semaglutide versus placebo in patients with heart failure and mildly reduced or preserved ejection fraction: a pooled analysis of the SELECT, FLOW, STEP-HFpEF, and STEP-HFpEF DM randomised trials. Lancet. (2024) 404:949–61. doi: 10.1016/s0140-6736(24)01643-x 39222642

[35] Pitt B Bhatt DL Szarek M Sun F Davies MJ Steg PG . The effect of the dual SGLT1 and 2 inhibitor sotagliflozin on cardiovascular mortality and hospital readmission rates for heart failure at 30 and 90 days post discharge in patients with type 2 diabetes hospitalized for worsening heart failure in the SOLOIST-WHF trial. Circulation. (2022) 11(8 Pt 1):879–89. doi: 10.1161/circ.146.suppl_1.10209

[36] McMurray JJV Ponikowski P Bolli GB Lukashevich V Kozlovski P Kothny W . Effects of vildagliptin on ventricular function in patients with type 2 diabetes mellitus and heart failure: a randomized placebo-controlled trial. (2018) 6:8–17. 10.1016/j.jchf.2017.08.004 29032139

[37] Petrie MC Verma S Docherty KF Inzucchi SE Anand I Belohlávek J . Effect of dapagliflozin on worsening heart failure and cardiovascular death in patients with heart failure with and without diabetes. JAMA. (2020) 323:1353–68. doi: 10.1001/jama.2020.1906 32219386 PMC7157181

[38] Pellicori P Ofstad AP Fitchett D Zeller C Wanner C George J . Early benefits of empagliflozin in patients with or without heart failure: findings from EMPA-REG OUTCOME. ESC Heart Fail. (2020) 7:3401–7. doi: 10.1002/ehf2.12891 32915523 PMC7754994

[39] Branch KRH Dagenais GR Avezum A Basile J Conget I Cushman WC . Dulaglutide and cardiovascular and heart failure outcomes in patients with and without heart failure: a post-hoc analysis from the REWIND randomized trial. (2022) 24:1805–12. doi: 10.1002/ejhf.2670 36073143

[40] Pitt B Bhatt DL Szarek M Cannon CP Leiter LA McGuire DK . Effect of sotagliflozin on early mortality and heart failure-related events: a post hoc analysis of SOLOIST-WHF. JACC Heart Fail. (2023) 11:879–89. doi: 10.1016/j.jchf.2023.05.026 37558385

[41] Kenny HC Abel ED . Heart failure in type 2 diabetes mellitus. Circ Res. (2019) 124:121–41. doi: 10.1161/circresaha.118.311371 30605420 PMC6447311

[42] Fernandez CJ Shetty S Pappachan JM . Diabetic cardiomyopathy: emerging therapeutic options. World J Diabetes. (2024) 15:1677–82. doi: 10.4239/wjd.v15.i8.1677 39192854 PMC11346103

[43] Cowie MR Fisher M . SGLT2 inhibitors: mechanisms of cardiovascular benefit beyond glycaemic control. Nat Rev Cardiol. (2020) 17:761–72. doi: 10.1038/s41569-020-0406-8 32665641

[44] Mavrakanas TA Tsoukas MA Brophy JM Sharma A Gariani K . SGLT-2 inhibitors improve cardiovascular and renal outcomes in patients with CKD: a systematic review and meta-analysis. Sci Rep. (2023) 13:15922. doi: 10.1038/s41598-023-42989-z 37741858 PMC10517929

[45] Alicic RZ Johnson EJ Tuttle KR . SGLT2 inhibition for the prevention and treatment of diabetic kidney disease: a review. Am J Kidney Dis. (2018) 72:267–77. doi: 10.1053/j.ajkd.2018.03.022 29866460

[46] Parsi S Shirsat PD Mahali LP Surani S Kashyap R . Sodium-glucose cotransporter 2 inhibitor in heart failure patients and their outcomes: a meta-analysis of randomized controlled trials. World J Cardiol. (2025) 17:109731. doi: 10.4330/wjc.v17.i10.109731 41181654 PMC12576562

[47] Cefalo CMA Cinti F Moffa S Impronta F Sorice GP Mezza T . Sotagliflozin, the first dual SGLT inhibitor: current outlook and perspectives. Cardiovasc Diabetol. (2019) 18:20. doi: 10.1186/s12933-019-0828-y 30819210 PMC6393994

[48] Bhatt DL Szarek M Steg PG Cannon CP Leiter LA McGuire DK . Sotagliflozin in patients with diabetes and recent worsening heart failure. N Engl J Med. (2021) 384:117–28. doi: 10.1056/nejmoa2030183 33200892

[49] Bhatt DL Szarek M Pitt B Cannon CP Leiter LA McGuire DK . Sotagliflozin in patients with diabetes and chronic kidney disease. N Engl J Med. (2021) 384:129–39. doi: 10.1056/nejmoa2030186 33200891

[50] Dominguez RJ Rieg T . What does sodium-glucose co-transporter 1 inhibition add: prospects for dual inhibition. Diabetes Obes Metab. (2019) 21 Suppl 2:43–52. doi: 10.1111/dom.13630 PMC6516085 31081587

[51] Drucker DJ . The benefits of GLP-1 drugs beyond obesity. Science. (2024) 385:258–60. doi: 10.1126/science.adn4128 39024455

[52] Sequeira V Theisen J Ermer KJ Oertel M Xu A Weissman D . Semaglutide normalizes increased cardiomyocyte calcium transients in a rat model of high fat diet-induced obesity. ESC Heart Fail. (2025) 12:1386–97. doi: 10.1002/ehf2.15152 PMC11911617 39482267

[53] Lincoff AM Brown-Frandsen K Colhoun HM Deanfield J Emerson SS Esbjerg S . Semaglutide and cardiovascular outcomes in obesity without diabetes. N Engl J Med. (2023) 389:2221–32. doi: 10.1056/nejmoa2307563 37952131

[54] Zheng Z Zong Y Ma Y Tian Y Pang Y Zhang C . Glucagon-like peptide-1 receptor: mechanisms and advances in therapy. Signal Transduct Target Ther. (2024) 9:234. doi: 10.1038/s41392-024-01931-z 39289339 PMC11408715

[55] Martins FL Bailey MA Girardi A . Endogenous activation of glucagon-like peptide-1 receptor contributes to blood pressure control: Role of proximal tubule Na(+)/H(+) exchanger isoform 3, renal angiotensin II, and insulin sensitivity. Hypertension. (2020) 76:839–48. doi: 10.1161/hypertensionaha.120.14868 32755467

[56] Zelniker TA Wiviott SD Raz I Im K Goodrich EL Furtado RHM . Comparison of the effects of glucagon-like peptide receptor agonists and sodium-glucose cotransporter 2 inhibitors for prevention of major adverse cardiovascular and renal outcomes in type 2 diabetes mellitus. Circulation. (2019) 139:2022–31. doi: 10.1161/circulationaha.118.038868 30786725

[57] Lam CSP Ramasundarahettige C Branch KRH Sattar N Rosenstock J Pratley R . Efpeglenatide and clinical outcomes with and without concomitant sodium-glucose cotransporter-2 inhibition use in type 2 diabetes: Exploratory analysis of the AMPLITUDE-O trial. Circulation. (2022) 145:565–74. doi: 10.1161/circulationaha.121.057934 34775781

[58] Packer M . Critical reanalysis of the mechanisms underlying the cardiorenal benefits of SGLT2 inhibitors and reaffirmation of the nutrient deprivation signaling/autophagy hypothesis. Circulation. (2022) 146:1383–405. doi: 10.1161/circulationaha.122.061732 36315602 PMC9624240

[59] Lo C Fei Y Cheung B . Cardiovascular outcomes in trials of new antidiabetic drug classes. Card Fail Rev. (2021) 7:e4. doi: 10.15420/cfr.2020.19 33747548 PMC7970669

[60] DeFronzo RA . Combination therapy with GLP-1 receptor agonist and SGLT2 inhibitor. Diabetes Obes Metab. (2017) 19:1353–62. doi: 10.1111/dom.12982 28432726 PMC5643008

[61] Nauck MA Quast DR Wefers J Meier JJ . GLP-1 receptor agonists in the treatment of type 2 diabetes - state-of-the-art. Mol Metab. (2021) 46:101102. doi: 10.1016/j.molmet.2020.101102 33068776 PMC8085572

[62] Wilson JM Nikooienejad A Robins DA Roell WC Riesmeyer JS Haupt A . The dual glucose-dependent insulinotropic peptide and glucagon-like peptide-1 receptor agonist, tirzepatide, improves lipoprotein biomarkers associated with insulin resistance and cardiovascular risk in patients with type 2 diabetes. Diabetes Obes Metab. (2020) 22:2451–9. doi: 10.1111/dom.14174 33462955 PMC7756479

[63] Gryglewska-Wawrzak K Kapłon-Cieślicka A Pawlak A Tomaszuk-Kazberuk A Rubiś P Niedziela J . Potential implications of body mass composition changes in heart failure patients in the era of SGLT2i, GLP-1 RA, and GIP/GLP-1 RA. Pharm (Basel). (2025) 18(11):1726. doi: 10.3390/ph18111726 41304969 PMC12655702

[64] Heise T DeVries JH Urva S Li J Pratt EJ Thomas MK . Tirzepatide reduces appetite, energy intake, and fat mass in people with type 2 diabetes. Diabetes Care. (2023) 46:998–1004. doi: 10.2337/dc22-1710 36857477 PMC10154650

[65] le Roux CW Hankosky ER Wang D Malik R Yu M Hickey A . Tirzepatide 10 and 15 mg compared with semaglutide 2.4 mg for the treatment of obesity: An indirect treatment comparison. Diabetes Obes Metab. (2023) 25:2626–33. doi: 10.1111/dom.15148 37344384

[66] Frías JP Davies MJ Rosenstock J Pérez Manghi FC Fernández Landó L Bergman BK . Tirzepatide versus semaglutide once weekly in patients with type 2 diabetes. N Engl J Med. (2021) 385:503–15. doi: 10.1056/NEJMoa2107519 34170647

[67] Augusto SJ Kaelber D Tang W . Real-world efficacy of tirzepatide in patients with heart failure without diabetes. Curr Probl Cardiol. (2025) 50:102998. doi: 10.1016/j.cpcardiol.2025.102998 39890046 PMC12090002

[68] Cavender MA Scirica BM Raz I Steg PG McGuire DK Leiter LA . Cardiovascular outcomes of patients in SAVOR-TIMI 53 by baseline hemoglobin A1c. Am J Med. (2016) 129:340–1. doi: 10.1016/j.amjmed.2015.09.022 26524706

[69] Cavallari I Bhatt DL Steg PG Leiter LA McGuire DK Mosenzon O . Causes and risk factors for death in diabetes: A competing-risk analysis from the SAVOR-TIMI 53 trial. J Am Coll Cardiol. (2021) 77:1837–40. doi: 10.1016/j.jacc.2021.02.030 33832610

[70] McAlister FA Zheng Y Westerhout CM Buse JB Standl E McGuire DK . Association between glycated haemoglobin levels and cardiovascular outcomes in patients with type 2 diabetes and cardiovascular disease: a secondary analysis of the TECOS randomized clinical trial. Eur J Heart Fail. (2020) 22:2026–34. doi: 10.1002/ejhf.1958 32621557

[71] Nauck MA McGuire DK Pieper KS Lokhnygina Y Strandberg TE Riefflin A . Sitagliptin does not reduce the risk of cardiovascular death or hospitalization for heart failure following myocardial infarction in patients with diabetes: observations from TECOS. Cardiovasc Diabetol. (2019) 18:116. doi: 10.1186/s12933-019-0921-2 31481069 PMC6719352

[72] Cavender MA White WB Jarolim P Bakris GL Cushman WC Kupfer S . Serial measurement of high-sensitivity troponin I and cardiovascular outcomes in patients with type 2 diabetes mellitus in the EXAMINE trial (Examination of cardiovascular outcomes with alogliptin versus standard of care). Circulation. (2017) 135:1911–21. doi: 10.1161/circulationaha.116.024632 28246236

[73] Lu CY . Observational studies: a review of study designs, challenges and strategies to reduce confounding. Int J Clin Pract. (2009) 63:691–7. doi: 10.1111/j.1742-1241.2009.02056.x 19392919

