# Supplementary material for: Network and pairwise meta-analysis of the effects of novel hypoglycemic agents on cardiorenal outcomes in patients with type 2 diabetes and heart failure
Source: Front Endocrinol (Lausanne). 2026 Oct 14;17:1955431. doi: 10.3389/fendo.2026.1955431 (PMC13713430; doi:10.3389/fendo.2026.1955431)
Supplement: Supplementary file 1 [file SupplementaryFile1.docx]

Supplementary Table S1. System retrieval strategy for Embase

**Supplementary table S1. Detailed search strategy for Embase.**

#1 "type 2 diabetes mellitus"/exp

#2 ("type 2 diabetes" OR T2DM OR diabetes mellitus OR NIDDM).ti,ab

#3 #1 OR #2

#4 "heart failure"/exp

#5 ("heart failure" OR "congestive heart failure" OR "cardiac failure" OR "heart decompensation").ti,ab

#6 #4 OR #5

#7 "sodium glucose co-transporter 2 inhibitor"/exp

#8 (canagliflozin OR dapagliflozin OR empagliflozin OR ertugliflozin OR sotagliflozin).ti,ab

#9 #7 OR #8

#10 "glucagon-like peptide 1 receptor agonist"/exp

#11 (exenatide OR lixisenatide OR liraglutide OR dulaglutide OR semaglutide).ti,ab

#12 #10 OR #11

#13 "dipeptidyl peptidase 4 inhibitor"/exp

#14 (sitagliptin OR vildagliptin OR saxagliptin OR linagliptin OR alogliptin OR teneligliptin).ti,ab

#15 #13 OR #14

#16 "GIP/GLP-1RA"/exp

#17 (tirzepatide).ti,ab

#18 #16 OR #17

#19 #9 OR #12 OR #15 OR #18

#20 #3 AND #6 AND #19

Supplementary Table S2. System retrieval strategy for Cochrane Central Register of Controlled Trials (CENTRAL)

**Supplementary table S2. Detailed search strategy for CENTRAL.**

#1 "diabetes mellitus, type 2" OR "type 2 diabetes" OR T2DM

#2 "heart failure" OR "congestive heart failure"

#3 "SGLT2 inhibitor*" OR "sodium-glucose transporter 2 inhibitor*" OR canagliflozin OR dapagliflozin OR empagliflozin OR ertugliflozin OR sotagliflozin

#4 "GLP-1 receptor agonist*" OR "glucagon-like peptide 1 receptor agonist*" OR exenatide OR lixisenatide OR liraglutide OR dulaglutide OR semaglutide

#5 "DPP-4 inhibitor*" OR "dipeptidyl peptidase 4 inhibitor*" OR sitagliptin OR vildagliptin OR saxagliptin OR linagliptin OR alogliptin OR teneligliptin

#6 "GIP/GLP-1 dual receptor agonist*" OR tirzepatide

#7 #3 OR #4 OR #5 OR #6

#8 #1 AND #2 AND #7

Supplementary Table S3. System retrieval strategy for Clinical Trials.gov

**Supplementary table S3. Detailed search strategy for Clinical Trials. gov.**

(type 2 diabetes OR T2DM) AND (heart failure OR congestive heart failure) AND (SGLT2 inhibitor* OR canagliflozin OR dapagliflozin OR empagliflozin OR ertugliflozin OR sotagliflozin OR GLP-1 receptor agonist* OR exenatide OR lixisenatide OR liraglutide OR dulaglutide OR semaglutide OR DPP-4 inhibitor* OR sitagliptin OR vildagliptin OR saxagliptin OR linagliptin OR alogliptin OR teneligliptin OR GIP/GLP-1 dual receptor agonist* OR tirzepatide)

Supplementary Table S4. Definition of Renal Composite Endpoint.

| **Study** | **Definition of Renal Composite Endpoint** |
| --- | --- |
| Gerasimos F et al. 2022 | First occurrence of chronic dialysis, renal transplantation, sustained reduction of ≥40% in eGFR, or sustained eGFR<15mL/min/1.73m^2^ for patients with baseline eGFR ≥30 mL/min/1.73m^2^ or <10mL/min/1.73m^2^ for patients with baseline eGFR <30mL/min/1.73m^2^. |
| Stefan DA et al. 2021 | First occurrence of chronic dialysis or renal transplant or a ≥40% decrease in eGFR or a sustained eGFR<15mL/min/1.73m^2^ for patients with baseline eGFR ≥30 mL/min/1.73m^2^ or <10mL/min/1.73m^2^ for patients with baseline eGFR <30mL/min/1.73m^2^. |
| Mark CP et al. 2020 | a 50% or greater sustained decline in eGFR(lasting ≥28days), renal failure(defined as a sustained eGFR <15 mL/min/1.73m², sustained dialysis treatment, or kidney transplant), or kidney-related death. |

Supplementary Table S5. Baseline sample sizes and raw event numbers of outcome endpoints in included studies.

| **Study** | **Treatment** | **sample size**  **(n)** | **HF-related composite endpoint(n)** | **Worsening of HF(n)** | **CV death(n)** | **All-cause death(n)** | **Hospitalization of HF(n)** | **Renal composite outcome(n)** |
| --- | --- | --- | --- | --- | --- | --- | --- | --- |
| Tomoya U (2021) | Canagliflozin | 42 | NR | NR | NR | NR | 1 | NR |
|  | Placebo | 40 | NR | NR | NR | NR | 1 | NR |
| Kelley RH (2022) | Dulaglutide | 421 | 87 | 55 | 53 | 84 | NR | NR |
|  | Placebo | 432 | 110 | 71 | 61 | 93 | NR | NR |
| Silvio E (2022) | dapagliflozin | 3150 | 1084 | 475 | 266 | 556 | 429 | NR |
|  | Placebo |  |  |  |  |  |  | NR |
| Pierpaolo P (2020) | Empagliflozin | 462 | 22 | NR | NR | 24 | 15 | NR |
|  | Placebo | 244 | 22 | NR | NR | 22 | 16 | NR |
| Gerasimos F (2022) | Empagliflozin | 1466 | 239 | NR | 120 | 234 | 250 | 75 |
|  | Placebo | 1472 | 291 | NR | 123 | 226 | 335 | 74 |
| Stefan DA (2021) | Empagliflozin | 927 | 200 | NR | 104 | NR | 221 | 22 |
|  | Placebo | 929 | 265 | NR | 113 | NR | 337 | 39 |
| Mikhail NK1 (2024) | Semaglutide | 167 | 40 | 29 | 17 | NR | NR | NR |
|  | Placebo | 158 | 40 | 29 | 19 | NR | NR | NR |
| Mikhail NK2 (2024) | Semaglutide | 310 | 9 | 7 | 2 | NR | NR | NR |
|  | Placebo | 306 | 20 | 18 | 4 | NR | NR | NR |
| Bertram P (2022) | Sotagliflozin | 290 | 31 | 25 | 9 | 9 | NR | NR |
|  | Placebo | 306 | 60 | 51 | 17 | 22 | NR | NR |
| Milton P (2025) | Tirzepatide | 182 | 18 | 13 | NR | 13 | NR | NR |
|  | Placebo | 189 | 29 | 27 | NR | 9 | NR | NR |
| John JV (2018) | Vildagliptin | 128 | 35 | 23 | 7 | 11 | NR | NR |
|  | Placebo | 126 | 31 | 22 | 4 | 4 | NR | NR |
| Mark CP (2020) | Dapagliflozin | 1075 | 215 | 7 | 121 | 143 | 138 | 18 |
|  | Placebo | 1064 | 271 | 11 | 148 | 178 | 172 | 24 |

Supplementary Table S6. Summary of effect estimates for all outcomes under random‑effects versus fixed‑effects models.

| **Outcome** | **Intervention** | **Random‑effects model[HR(95%CI)]** | **Sensitivity analysis Fixed‑effects model[HR(95%CI)]** |
| --- | --- | --- | --- |
| HF-related composite endpoint | SGLT2i vs GLP-1RA | 0.976(0.761, 1.250) | 0.977 (0.767, 1.245) |
|  | SGLT2i vs GIP/GLP-1 | 1.171(0.639, 2.146) | 1.174 (0.644, 2.141) |
|  | SGLT2i vs DPP-4i | 0.675(0.413, 1.104) | 0.677 (0.416, 1.101) |
|  | SGLT2i vs Placebo | 0.749 (0.684, 0.821) | 0.751 (0.688, 0.819) |
|  | GLP-1RA vs GIP/GLP-1 | 1.200(0.632, 2.278) | 1.202 (0.636, 2.268) |
|  | GLP-1RA vs DPP-4i | 0.692(0.405, 1.182) | 0.693 (0.408, 1.177) |
|  | GLP-1RA vs Placebo | 0.768(0.610, 0.967) | 0.769 (0.613, 0.964) |
|  | GIP/GLP-1 vs DPP-4i | 0.577(0.267, 1.244) | 0.577 (0.269, 1.238) |
|  | GIP/GLP-1 vs Placebo | 0.640(0.352, 1.164) | 0.640 (0.353, 1.160) |
|  | DPP-4i vs Placebo | 1.110(0.684, 1.800) | 1.110 (0.687, 1.793) |
| Worsening of HF | SGLT2i vs GLP-1RA | 0.975(0.618, 1.536) | 1.040 (0.755, 1.433) |
|  | SGLT2i vs GIP/GLP-1 | 1.460(0.655, 3.247) | 1.585 (0.793, 3.175) |
|  | SGLT2i vs DPP-4i | 0.694(0.334, 1.443) | 0.755 (0.409, 1.391) |
|  | SGLT2i vs Placebo | 0.715(0.532, 0.961) | 0.777 (0.658, 0.917) |
|  | GLP-1RA vs GIP/GLP-1 | 1.497(0.659, 3.401) | 1.524 (0.738, 3.155) |
|  | GLP-1RA vs DPP-4i | 0.712(0.335, 1.513) | 0.726 (0.379, 1.389) |
|  | GLP-1RA vs Placebo | 0.734(0.519, 1.037) | 0.747 (0.568, 0.983) |
|  | GIP/GLP-1 vs DPP-4i | 0.476(0.175, 1.295) | 0.476 (0.195, 1.163) |
|  | GIP/GLP-1 vs Placebo | 0.490(0.233, 1.032) | 0.490 (0.250, 0.960) |
|  | DPP-4i vs Placebo | 1.030(0.527, 2.012) | 1.030 (0.572, 1.856) |
| CV death | SGLT2i vs GLP-1RA | 1.028(0.730, 1.445) | 1.038 (0.738, 1.460) |
|  | SGLT2i vs DPP-4i | 0.506(0.147, 1.745) | 0.512 (0.148, 1.764) |
|  | SGLT2i vs Placebo | 0.870(0.770, 0.983) | 0.880 (0.778, 0.995) |
|  | GLP-1RA vs DPP-4i | 0.493(0.138, 1.759) | 0.493 (0.138, 1.761) |
|  | GLP-1RA vs Placebo | 0.847(0.616, 1.165) | 0.847 (0.616, 1.165) |
|  | DPP-4i vs Placebo | 1.720(0.502, 5.898) | 1.720 (0.502, 5.898) |
| All-cause death | SGLT2i vs GLP-1RA | 0.901(0.549, 1.477) | 0.972 (0.712, 1.326) |
|  | SGLT2i vs GIP/GLP-1 | 0.552(0.216, 1.414) | 0.596 (0.253, 1.407) |
|  | SGLT2i vs Placebo | 0.829(0.674, 1.019) | 0.894 (0.806, 0.991) |
|  | GLP-1RA vs GIP/GLP-1 | 0.613(0.221, 1.704) | 0.613 (0.249, 1.511) |
|  | GLP-1RA vs Placebo | 0.920(0.587, 1.442) | 0.920 (0.686, 1.233) |
|  | GIP/GLP-1 vs Placebo | 1.500(0.599, 3.756) | 1.500 (0.640, 3.518) |
| Hospitalization of HF | SGLT2i vs Placebo | 0.74(0.66, 0.83) | 0.74(0.66, 0.83) |
| Renal composite outcome | SGLT2i vs Placebo | 0.76(0.51, 1.14) | 0.82(0.64, 1.06) |

Supplementary Table S7. Summary of effect estimates after excluding the SOLOIST‑WHF in random‑effects models.

| **Outcome** | **Intervention** | **Primary analysisIncluding sotagliflozin(HR(95%CI))** | **Sensitivity analysisExcluding SOLOIST‑WHF(HR(95%CI))** |
| --- | --- | --- | --- |
| HF-related composite endpoint | SGLT2i vs GLP-1RA | 0.976(0.761, 1.250) | 0.991 (0.777, 1.264) |
|  | SGLT2i vs GIP/GLP-1 | 1.171(0.639, 2.146) | 1.191 (0.652, 2.174) |
|  | SGLT2i vs DPP-4i | 0.675(0.413, 1.104) | 0.686 (0.422, 1.117) |
|  | SGLT2i vs Placebo | 0.749 (0.684, 0.821) | 0.762 (0.697, 0.833) |
|  | GLP-1RA vs GIP/GLP-1 | 1.200(0.632, 2.278) | 1.202 (0.636, 2.268) |
|  | GLP-1RA vs DPP-4i | 0.692(0.405, 1.182) | 0.693 (0.408, 1.177) |
|  | GLP-1RA vs Placebo | 0.768(0.610, 0.967) | 0.769 (0.613, 0.964) |
|  | GIP/GLP-1 vs DPP-4i | 0.577(0.267, 1.244) | 0.577 (0.269, 1.238) |
|  | GIP/GLP-1 vs Placebo | 0.640(0.352, 1.164) | 0.640 (0.353, 1.160) |
|  | DPP-4i vs Placebo | 1.110(0.684, 1.800) | 1.110 (0.687, 1.793) |
| Worsening of HF | SGLT2i vs GLP-1RA | 0.975(0.618, 1.536) | 1.099 (0.793, 1.524) |
|  | SGLT2i vs GIP/GLP-1 | 1.460(0.655, 3.247) | 1.678 (0.836, 3.367) |
|  | SGLT2i vs DPP-4i | 0.694(0.334, 1.443) | 0.797 (0.431, 1.475) |
|  | SGLT2i vs Placebo | 0.715(0.532, 0.961) | 0.822 (0.688, 0.981) |
|  | GLP-1RA vs GIP/GLP-1 | 1.497(0.659, 3.401) | 1.524 (0.737, 3.155) |
|  | GLP-1RA vs DPP-4i | 0.712(0.335, 1.513) | 0.726 (0.379, 1.389) |
|  | GLP-1RA vs Placebo | **0.734(0.519, 1.037)** | **0.747 (0.568, 0.983)** |
|  | GIP/GLP-1 vs DPP-4i | 0.476(0.175, 1.295) | 0.476 (0.195, 1.163) |
|  | GIP/GLP-1 vs Placebo | **0.490(0.233, 1.032)** | **0.490 (0.250, 0.960)** |
|  | DPP-4i vs Placebo | 1.030(0.527, 2.012) | 1.030 (0.572, 1.856) |
| CV death | SGLT2i vs GLP-1RA | 1.028(0.730, 1.445) | 1.028 (0.731, 1.445) |
|  | SGLT2i vs DPP-4i | 0.506(0.147, 1.745) | 0.506 (0.147, 1.745) |
|  | SGLT2i vs Placebo | 0.870(0.770, 0.983) | 0.870 (0.770, 0.983) |
|  | GLP-1RA vs DPP-4i | 0.493(0.138, 1.759) | 0.493 (0.138, 1.761) |
|  | GLP-1RA vs Placebo | 0.847(0.616, 1.165) | 0.847 (0.616, 1.165) |
|  | DPP-4i vs Placebo | 1.720(0.502, 5.898) | 1.720 (0.502, 5.898) |
| All-cause death | SGLT2i vs GLP-1RA | 0.901(0.549, 1.477) | 0.952 (0.615, 1.471) |
|  | SGLT2i vs GIP/GLP-1 | 0.552(0.216, 1.414) | 0.584 (0.235, 1.451) |
|  | SGLT2i vs Placebo | 0.829(0.674, 1.019) | 0.876 (0.731, 1.049) |
|  | GLP-1RA vs GIP/GLP-1 | 0.613(0.221, 1.704) | 0.613 (0.231, 1.629) |
|  | GLP-1RA vs Placebo | 0.920(0.587, 1.442) | 0.920 (0.619, 1.368) |
|  | GIP/GLP-1 vs Placebo | 1.500(0.599, 3.756) | 1.500 (0.614, 3.665) |

Supplementary Figure 1. Egger’s test.

Supplementary Figure 2. Leave-one-out sensitivity analysis.
